# Supplementary material for: Social threat avoidance depends on action-outcome predictability
Source: Commun Psychol. 2024 Oct 26;2:100. doi: 10.1038/s44271-024-00152-y (PMC11512816; doi:10.1038/s44271-024-00152-y)
Supplement: Supplementary file 2 — Supplementary Information [file 44271_2024_152_MOESM2_ESM.pdf]

Supplementary Information

Social threat avoidance depends on action-outcome predictability

Matteo Sequestro[1*], Jade Serfaty[1], and Julie Grèzes[1#*], Rocco Mennella[1,2#]

[1] Cognitive and Computational Neuroscience Laboratory (LNC2), Inserm U960, Department of Cognitive Studies, École Normale Supérieure, PSL University, 29 rue d'Ulm, 75005, Paris, France.

[2] Laboratory of the Interactions between Cognition Action and Emotion (LICAÉ, EA2931), UFR STAPS, Université Paris Nanterre, 200 avenue de La République, 92001 Nanterre Cedex, France.

[#] These authors jointly supervised this work

[*]**Corresponding authors**

Matteo Sequestro [0009-0007-2670-7505]: matteo.sequestro@gmail.com

Julie Grèzes [0000-0002-5066-9154]: julie.grezes@ens.psl.eu

Supplementary Methods 1: Inclusion Criteria

Participants had to:

1. Be aged between 18 and 35 years old.
2. Be able to speak French fluently.
3. Not have major vision or hearing problems.
4. Not wear glasses or wear contact lenses on the day of the study.
5. Not be pregnant.
6. Not be claustrophobic.
7. Not suffer from postural instability or vestibular disorders.
8. Not suffer from motion sickness.
9. Not suffer from oculomotor disorders (strabismus, convergence disorder, anisophoria, anisometropia (asymmetrical refraction disorder), relative amblyopia (difference in visual perception between the two eyes), uncorrected ametropia, ocular pathologies or anomalies.
10. Not suffer from migraines.
11. Not have any affective disorders (depression, anxiety disorders, etc.) or behavioral disorders (anorexia, bulimia, addiction, etc.).
12. Not have medical history of neurological or psychiatric problems (epilepsy, depression, schizophrenia, apraxia, head trauma, etc.).
13. Not take any neurological, cardiac, or psychiatric medication.
14. Be willing to not consume alcohol the night before the study.
15. Be willing to not take any narcotics in the week preceding the study (marijuana, cocaine, ecstasy, MDMA, ketamine, etc.).
16. Agree to their data being made available anonymously to the scientific community criteria.

Furthermore, participants were asked not to drink alcohol the night before the experiment and not to assume drugs the week before the experiment.

From Experiment 2, participants were also required not to suffer from any cardiovascular disorders and not to have smoked tobacco for at least two hours before the experiment.

Supplementary Methods 2: Stimuli Creation

Selection and Morphing of Facial Emotions

20 actors (10 females) were selected from the Radboud Face Database[1]. For each, two expressions were selected (neutral and angry). Thus, the selected faces consisted of 10 actors x 2 sexes x 2 expressions (N_{total} = 40). Lately, 4 avatars (a dyad for each sex) were discarded due to their bad quality in virtual reality, leaving a final amount of 36 faces. In order to create 3D head models from the 2D face stimuli, we used the DECA model (Detailed Expression Capture and Animation[2]). The model projects a 2D image to a 3D model surface by "UV mapping". Spurious features of the UV maps thus obtained were removed using a custom black mask. The Meshlab software[3] was then used to align the 3D reconstructed morphs for stimuli display. Vertical alignment was made by keeping noses in line across actors and expressions, while horizontal alignment was made by keeping the eyes in line. The aligned 3D morphs were then exported to the Blender software (Blender Foundation), where an eye-bulge correction was performed using the "mesh" and "UV Map" editing tools in case of spurious reconstructions.

3D face-body merge

Four 3D human body models (two males, two females) presenting grey scale-colored clothes and simple hairstyles were purchased from the RenderPeople website. The validated 3D stimuli previously described were merged with these models in Blender using the "slicing", "object joining" and "vertex stitching" editing tools. We also used the "smoothing" tool to sculpt the uneven vertices and edges to frame a realistic body with the new face. A texture color correction was conducted to ensure smooth transition between body and face colors. Then, we created meta-rigs (i.e., a simulation of bones and articulation for each body) in order to control the posture in the Virtual Environment. Finally, we added a basic walk animation for the individuals entering and exiting the elevators. Such idle movements, consisting in small body oscillations, provide a more naturalistic view of the individuals[4].

Supplementary Methods 3: Bayesian hierarchical GLM model fitting and selection

Model fitting

Bayesian Hierarchical binomial Generalized Linear Models (or Generalized Linear Mixed Effect Model or GLMM) have been fitted using the R package *brms*[5] (v2.16.1). The package performs parameter estimation using Markov Chain Monte Carlo (MCMC) sampling. In all experiments and for each model three chains were run in parallel with 10,000 iterations each, 2,000 of which have been used as a warm-up period. In the most complex model we fitted single-trial avoidance responses as a function of the Condition (Unpredictable = 0 vs. Predictable = 1), the RTs (grand average centered) and their interaction. Individual participants have been introduced as a random effect keeping a maximal random slope structure. We used the following priors to fit the model:

β_0	~	N(0, 2.5)
$\beta_{predictors}$	~	N(0, 2.5)
cor	~	lkj(2)
SD	~	exponential(2)

Where β_0 is the prior for the intercept, $\beta_{predictors}$ is the prior for the slope of predictors, N is the gaussian distribution, cor is the correlation matrix, lkj is the Lewandowski-Kurowicka-Joe distribution. Similar priors have been previously suggested in the case of GLMMs[6].

For all experiments, we performed a prior sensitivity analysis by refitting the best fitting models with a distinct set of priors. Firstly, we refitted the models by using brms' default priors, which provide parameter estimates similar to those provided by traditional frequentist non-Bayesian methods. These priors consist of flat priors for β_0 and $\beta_{predictors}$, a lkj(1) prior for the correlation matrix and a StudentT(3, 0, 2.5) prior for the standard deviation parameters. We will refer to this set of priors as "priors set 2".

Secondly, we refitted the models using the following set of priors: $\beta_0 \sim N(0.405, 1)$, $\beta_{predictors} \sim N(0, 1)$, cor ~ lkj(2), and SD ~ HalfCauchy(0,1). We will refer to this set of priors as "priors set

3". Note that using a N(0.405, 1) for the intercept reflects a prior belief that participants will present a proportion of avoidance responses of about 60% in the unpredictable condition, as the priors is expressed on a logit scale and as the condition variable in the model is treatment coded (Unpredictable = 0, Predictable = 1), so the intercept represents the unpredictable condition. The 60% proportion for avoidance choices is coherent with what we have found in previous studies using a similar free-choice approach-avoidance task[7,8], which did not manipulate the predictability of the action-outcome. Therefore, since the prior for predictors is centered at zero, this priors set can be considered skeptical of our hypothesis that participants would avoid more in the predictable condition compared to the unpredictable one. However, it is not skeptical of the fact that participants will prefer to avoid the angry avatar, unlike our main priors set. Finally, a HalfCauchy(0, 1) for the standard deviation parameter has been previously suggested for mixed effect models[6].

Experiment 1

Model selection

Stepwise model selection has been performed by running several models with an increasing number of predictors and comparing their WAIC indexes. For Experiment 1 the best fitting model has been found to be the one including the Condition and RTs effect as predictors, as well as their interaction (*Table S1*).

Table S1: *Regression Model comparison for Experiment 1.*

model	WAIC	SE
Inter.	4367.94	52.81
Inter. + Cond.	4300.26	54.22
Inter. + RTs	4330.14	54.19
Inter. + Cond. + RTs	4288.70	55.02
**Inter. + Cond. + RTs + Cond.:RTs**	**4288.03**	**55.11**

Note: WAIC: Widely Applicable Information Criterion. SE: Standard Error. Inter.: Intercept. Cond.: Condition. RTs: Response Times. Bold: Best fitting model (i.e. lowest WAIC).

Model diagnostic

Chain Convergence has been visually inspected and verified. All chains presented a 'hairy caterpillar' shape. In *Tables S2 and S3* we provide R-hat and Effective Sample Size (ESS) statistics showing that chains properly mixed.

Table S2: *Experiment 1 model results and diagnostic for each parameter.*

	Est.	Est.Err.	95%CrI-low	95%CrI-up	$p_{>0}$	$p_{<0}$	BF_{01}	BF_{10}	R-hat	ESS
Inter.	.703	.096	.519	.896	1	0	0	5.645e+14	1	7538
Cond.	.475	.114	.253	.699	1	0	.008	128.297	1	10261
RTs	1.050	.249	.571	1.544	1	0	.003	288.812	1	10653
Cond.:RTs	.291	.377	-.467	1.013	.785	.215	4.841	.207	1.001	9028

Note: Inter: Intercept. Cond.: Condition effect. RTs: Response Times effect Est.: Estimate. Est.Err.: Estimate Error. 95%CrI-low and -up: lower and upper boundary of the 95% Credible Interval. $p_{>0}$ and $p_{<0}$: proportion of posterior samples greater and lower than zero. BF_{01} and BF_{10}: Bayes Factor for the parameter to be equal or different from zero. Rhat: R-hat potential scale reduction factor. ESS: Effective Sample Size.

Table S3: *Experiment 1 model random effect table*

	Estimate	Est.Error	95%CrI-low	95%CrI-up	R-hat	ESS
sd(Inter.)	.573	.089	.410	.763	1	8342
sd(Cond.)	.502	.145	.186	.775	1.001	2917
sd(RTs)	.599	.355	.026	1.316	1	3180
sd(Cond.:RTs)	.839	.526	.032	1.892	1.001	3288
cor(Inter.,Cond.)	-.081	.239	-.493	.428	1	6948
cor(Inter., RTs)	.169	.322	-.499	.741	1	12032
cor(Cond., RTs)	-.025	.326	-.644	.618	1	9669
cor(Inter., Cond.:RTs)	-.216	.319	-.753	.485	1	10826
cor(Cond., Cond.:RTs)	.036	.336	-.609	.677	1	11041
cor(RTs, Cond.:RTs)	.086	.355	-.613	.731	1	9076

Note: sd: standard deviation. cor: correlation. Inter: Intercept. Cond: Condition. RTs: Response Times. Est.Error: Estimate Error. 95%CrI-low and -up: lower and upper boundary of the 95% Credible Interval. Rhat: R-hat potential scale reduction factor. ESS: Effective Sample Size.

Prior sensitivity analysis

As shown in *Tables S4* and *S5*, refitting the model using priors set 2 (brms' default priors) did not substantially influence our results nor interpretations.

Table S4: *Experiment 1 model results with priors set 2*

	Est.	Est.Err.	95%CrI-low	95%CrI-up	$p_{>0}$	$p_{<0}$	R-hat	ESS
Inter.	.708	.098	.516	.906	1	0	1	15274
Cond.	.506	.121	.274	.752	1	0	1	18361
RTs	1.081	.268	.564	1.618	1	0	1	18806
Cond.:RTs	.228	.424	-.632	1.040	.710	.290	1	16659

Note: Inter: Intercept. Cond.: Condition effect. RTs: Response Times effect. Est.: Estimate. Est.Err.: Estimate Error. 95%CrI-low and -up: lower and upper boundary of the 95% Credible Interval. $p_{>0}$ and $p_{<0}$: proportion of posterior samples greater and lower than zero. BF_{01} and BF_{10}: Bayes Factor for the parameter to be equal or different from zero. Rhat: R-hat potential scale reduction factor. ESS: Effective Sample Size.

Table S5*: Experiment 1 model random effect table with priors set 2*

	Estimate	Est.Error	95%CrI-low	95%CrI-up	R-hat	ESS
sd(Inter.)	.592	.095	.422	.795	1	11354
sd(Cond.)	.532	.150	.225	.825	1	5085
sd(RTs)	.802	.377	.080	1.538	1	4950
sd(Cond.:RTs)	1.309	.558	.178	2.382	1.002	5484
cor(Inter.,Cond.)	-.068	.256	-.518	.467	1	11810
cor(Inter., RTs)	.291	.345	-.463	.861	1	14577
cor(Cond., RTs)	-.082	.356	-.717	.655	1	11831
cor(Inter., Cond.:RTs)	-.358	.301	-.845	.333	1	12846
cor(Cond., Cond.:RTs)	.043	.359	-.647	.723	1	12306
cor(RTs, Cond.:RTs)	.061	.389	-.679	.789	1	9871

Note: sd: standard deviation. cor: correlation. Inter: Intercept. Cond: Condition. RTs: Response Times. Cond:RTs: Condition by Response Times interaction. Est.Error: Estimate Error. 95%CrI-low and -up: lower and upper boundary of the 95% Credible Interval. Rhat: R-hat potential scale reduction factor. ESS: Effective Sample Size.

Similarly, as shown in *Tables S6* and *S7*, refitting the model using priors set 3 did not substantially influence our results nor interpretations.

Table S6: *Experiment 1 model results with priors set 3*

	Est.	Est.Err.	95%CrI-low	95%CrI-up	$p_{>0}$	$p_{<0}$	BF_{01}	BF_{10}	R-hat	ESS
Inter.	.700	.098	.511	.894	1	0	0	2.787e+15	1	11351
Cond.	.494	.118	.263	.729	1	0	.001	1.148e+3	1	15225
RTs	1.018	.245	.540	1.497	1	0	0	2.847e+3	1	16910

Cond.:RTs	.312	.369	-.43	1.033	.805	.195	2.137	.047	1	15963

Note: Inter: Intercept. Cond.: Condition effect. RTs: Response Times effect. Est.: Estimate. Est.Err.: Estimate Error. 95%CrI-low and -up: lower and upper boundary of the 95% Credible Interval. $p_{>0}$ and $p_{<0}$: proportion of posterior samples greater and lower than zero. BF_{01} and BF_{10}: Bayes Factor for the parameter to be equal or different from zero. Rhat: R-hat potential scale reduction factor. ESS: Effective Sample Size.

***Table S7**: Experiment 1 model random effect table with priors set 3*

	Estimate	Est.Error	95%CrI-low	95%CrI-up	R-hat	ESS
sd(Inter.)	.588	.093	.419	.786	1	9744
sd(Cond.)	.517	.143	.219	.798	1	4596
sd(RTs)	.714	.362	.061	1.432	1.002	4634
sd(Cond.:RTs)	1.128	.548	.111	2.197	1.001	4835
cor(Inter.,Cond.)	-.073	.240	-.500	.435	1	10426
cor(Inter., RTs)	.188	.318	-.468	.750	1	14898
cor(Cond., RTs)	-.043	.317	-.632	.591	1	12907
cor(Inter., Cond.:RTs)	-.262	.288	-.754	.369	1	15857
cor(Cond., Cond.:RTs)	.043	.322	-.580	.563	1	13088
cor(RTs, Cond.:RTs)	.083	.345	-.589	.722	1	10402

Note: sd: standard deviation. cor: correlation. Inter: Intercept. Cond: Condition. RTs: Response Times. Est.Error: Estimate Error. 95%CrI-low and -up: lower and upper boundary of the 95% Credible Interval. R-hat: R-hat potential scale reduction factor. ESS: Effective Sample Size.

Experiment 2

Model selection

For Experiment 2 the best fitting model has been found to be the one including the Condition and RT effect as predictors, as well as their interaction (*Table S8*).

***Table S8:** Regression Model comparison for Experiment 2.*

model	WAIC	SE
Inter.	2427.79	26.88
Inter. + Cond.	2393.75	28.90
Inter. + RTs	2411.37	28.20
Inter. + Cond. + RTs	2383.18	29.56
**Inter. + Cond. + RTs + Cond.:RTs**	**2380.75**	**29.88**

Note: WAIC: Widely Applicable Information Criterion. SE: Standard Error. Inter.: Intercept. Cond.: Condition. RTs: Response Times. Bold: Best fitting model (i.e. lowest WAIC).

Model diagnostic

Chain Convergence has been visually inspected and verified. All chains presented a 'hairy caterpillar' shape. In *Tables S9* and *S10* we provide R-hat and Effective Sample Size (ESS) statistics showing that chains properly mixed.

Table S9: *Experiment 2 model results and diagnostic for each parameter.*

	Est.	Est.Err.	95%CrI-low	95%CrI-up	$p_{>0}$	$p_{<0}$	BF_{01}	BF_{10}	R-hat	ESS
Inter.	.416	.107	.206	.629	1	0	.033	29.969	1	14030
Cond.	.303	.120	.065	.539	.993	.007	1.079	.927	1	22454
RTs	.426	.299	-.159	1.019	.924	.076	3.044	.328	1	16403
Cond.:RTs	1.476	.376	.726	2.195	1	0	.005	182.234	1	19657

Note: Inter: Intercept. Cond.: Condition effect. RTs: Response Times effect. Est.: Estimate. Est.Err.: Estimate Error. 95%CrI-low and -up: lower and upper boundary of the 95% Credible Interval. $p_{>0}$ *and* $p_{<0}$*: proportion of posterior samples greater and lower than zero.* BF_{01} *and* BF_{10}*: Bayes Factor for the parameter to be equal or different from zero. R-hat: R-hat potential scale reduction factor. ESS: Effective Sample Size.*

Table S10*: Experiment 2 model random effect table*

	Estimate	Est.Error	95%CrI-low	95%CrI-up	R-hat	ESS
sd(Inter.)	.406	.104	.218	.626	1	9664
sd(Cond.)	.301	.169	.017	.644	1	5003
sd(RTs)	.722	.326	.080	1.371	1	6314
sd(Cond.:RTs)	.367	.312	.012	1.147	1	12507
cor(Inter.,Cond.)	-.346	.329	-.831	.4233	1	12102
cor(Inter., RTs)	.445	.285	-.222	.872	1	13762
cor(Cond., RTs)	-.113	.347	-.731	.590	1	14251
cor(Inter., Cond.:RTs)	-.037	.370	-.722	.673	1	30341
cor(Cond., Cond.:RTs)	.115	.380	-.645	.769	1	24773
cor(RTs, Cond.:RTs)	-.086	.376	-.748	.660	1	26957

Note: sd: standard deviation. cor: correlation. Inter: Intercept. Cond: Condition. RTs: Response Times. Est.Error: Estimate Error. 95%CrI-low and -up: lower and upper boundary of the 95% Credible Interval. R-hat: R-hat potential scale reduction factor. ESS: Effective Sample Size.

Prior sensitivity analysis

As shown in *Tables S11* and *S12*, refitting the model using priors set 2 (brms' default priors) did not substantially influence our results nor interpretations.

Table S11: *Experiment 2 model results with priors set 2*

	Est.	Est.Err.	95%CrI-low	95%CrI-up	$p_{>0}$	$p_{<0}$	R-hat	ESS
Inter.	.418	.120	.182	.657	.999	.001	1	8553
Cond.	.289	.137	.015	.556	.980	.020	1	12559
RTs	.487	.346	-.178	1.193	.926	.074	1	9057
Cond.:RTs	1.443	.430	.568	2.262	.999	.001	1	12511

Note: Inter: Intercept. Cond.: Condition effect. RTs: Response Times effect. Est.: Estimate. Est.Err.: Estimate Error. 95%CrI-low and -up: lower and upper boundary of the 95% Credible Interval. $p_{>0}$ and $p_{<0}$: proportion of posterior samples greater and lower than zero. BF_{01} and BF_{10}: Bayes Factor for the parameter to be equal or different from zero. R-hat: R-hat potential scale reduction factor. ESS: Effective Sample Size.

Table S12*: Experiment 2 model random effect table with priors set 2*

	Estimate	Est.Error	95%CrI-low	95%CrI-up	R-hat	ESS
sd(Inter.)	.466	.116	.258	.716	1.001	8918
sd(Cond.)	.405	.180	.053	.763	1	4536
sd(RTs)	1.056	.383	.329	1.852	1	7195
sd(Cond:RTs)	.792	.503	.042	1.890	1	7026
cor(Inter.,Cond.)	-.518	.305	-.920	.267	1	8589
cor(Inter., RTs)	.617	.253	.001	.949	1	9831
cor(Cond., RTs)	-.282	.357	-.858	.494	1	9865
cor(Inter., Cond.:RTs)	-.238	.409	-.882	.649	1	12833
cor(Cond., Cond.:RTs)	.343	.404	-.588	.915	1	10519
cor(RTs, Cond.:RTs)	-.317	.409	-.899	.626	1	12050

Note: sd: standard deviation. cor: correlation. Inter: Intercept. Cond: Condition. RTs: Response Times. Est.Error: Estimate Error. 95%CrI-low and -up: lower and upper boundary of the 95% Credible Interval. R-hat: R-hat potential scale reduction factor. ESS: Effective Sample Size.

Similarly, as shown in *Tables S13* and *S14*, refitting the model using priors set 3 did not substantially influence our results nor interpretations.

Table S13: *Experiment 2 model results with priors set 3*

	Est.	Est.Err.	95%CrI-low	95%CrI-up	$p_{>0}$	$p_{<0}$	BF_{01}	BF_{10}	R-hat	ESS
Inter.	.413	.110	.200	.633	1	0	.019	52.631	1	10856
Cond.	.299	.126	.051	.547	.990	.010	.551	1.814	1	15277
RTs	.490	.302	-.089	1.086	.951	.049	.984	1.016	1	13278

Cond.:RTs	1.343	.375	.580	2.063	.999	.001	.014	73.378	1	13882

Note: Inter: Intercept. Cond.: Condition effect. RTs: Response Times effect. Est.: Estimate. Est.Err.: Estimate Error. 95%CrI-low and -up: lower and upper boundary of the 95% Credible Interval. $p_{>0}$ and $p_{<0}$: proportion of posterior samples greater and lower than zero. BF_{01} and BF_{10}: Bayes Factor for the parameter to be equal or different from zero. R-hat: R-hat potential scale reduction factor. ESS: Effective Sample Size.

Table S14: *Experiment 2 model random effect table with priors set 3*

	Estimate	Est.Error	95%CrI-low	95%CrI-up	R-hat	ESS
sd(Inter.)	.430	.109	.235	.661	1	8503
sd(Cond.)	.358	.174	.031	.702	1.001	4377
sd(RTs)	.862	.342	.174	1.562	1	6819
sd(Cond:RTs)	.545	.399	.021	1.479	1.001	8626
cor(Inter.,Cond.)	-.394	.306	-.841	.337	1	9976
cor(Inter., RTs)	.477	.269	-.149	.879	1	11138
cor(Cond., RTs)	-.142	.334	-.738	.539	1	10741
cor(Inter., Cond.:RTs)	-.098	.368	-.751	.637	1	19021
cor(Cond., Cond.:RTs)	.188	.368	-.582	.804	1	15801
cor(RTs, Cond.:RTs)	-.163	.375	-.794	.608	1	16758

Note: sd: standard deviation. cor: correlation. Inter: Intercept. Cond: Condition. RTs: Response Times. Est.Error: Estimate Error. 95%CrI-low and -up: lower and upper boundary of the 95% Credible Interval. R-hat: R-hat potential scale reduction factor. ESS: Effective Sample Size.

Experiment 3

Model Selection

For Experiment 3 the best fitting model was the one including the Condition and RT effect as predictors, as well as their interaction (*Table S15*).

Table S15: *Regression Model comparison for Experiment 3.*

model	WAIC	SE
Inter.	4928.97	34.32
Inter. + Cond.	4895.15	36.12
Inter. + RTs	4909.65	35.77
Inter. + Cond. + RTs	4875.97	37.24
**Inter. + Cond. + RTs + Cond.:RTs**	**4864.74**	**37.82**

Note: WAIC: Widely Applicable Information Criterion. SE: Standard Error. Inter.: Intercept. Cond.: Condition. RTs: Response Times. Bold: Best fitting model (i.e. lowest WAIC).

Model diagnostic

Chain Convergence has been visually inspected and verified. All chains presented a 'hairy caterpillar' shape. In *Tables S16* and *S17* we provide R-hat and Effective Sample Size (ESS) statistics showing that chains properly mixed.

Table S16: *Experiment 3 model results and diagnostic for each parameter.*

	Est.	Est.Err.	95%CrI-low	95%CrI-up	$p_{>0}$	$p_{<0}$	BF_{01}	BF_{10}	R-hat	ESS
Inter.	.301	.076	.152	.450	1	0	.012	81.860	1	12906
Cond.	.188	.087	.019	.360	.986	.015	2.691	.372	1	16975
RTs	.714	.247	.229	1.198	.998	.002	.161	6.226	1	14184
Cond.:RTs	1.115	.331	.458	1.756	.999	.001	.028	35.638	1	17577

Note: Inter: Intercept. Cond.: Condition effect. RTs: Response Times effect. Est.: Estimate. Est.Err.: Estimate Error. 95%CrI-low and -up: lower and upper boundary of the 95% Credible Interval. p>0 and p<0: proportion of posterior samples greater and lower than 0. BF01 and BF10: Bayes Factor for the parameter to be equal or different from 0. R-hat: R-hat potential scale reduction factor. ESS: Effective Sample Size.

Table S17: *Experiment 3 model random effect table*

	Estimate	Est.Error	95%CrI-low	95%CrI-up	R-hat	ESS
sd(Inter.)	.399	.075	.264	.559	1	8471
sd(Cond.)	.261	.140	.016	.533	1.002	2752
sd(RTs)	.771	.287	.164	1.330	1	5897
sd(Cond.:RTs)	1.116	.480	.114	2.004	1.001	3472
cor(Inter.,Cond.)	-.210	.314	-.727	.492	1.001	8715
cor(Inter., RTs)	.619	.222	.075	.919	1	7780
cor(Cond., RTs)	-.050	.322	-.657	.589	1	15227
cor(Inter., Cond:RTs)	-.231	.279	-.726	.354	1	13975
cor(Cond., Cond:RTs)	.337	.324	-.424	.833	1.001	5548
cor(RTs, Cond:RTs)	-.256	.312	-.756	.449	1	7680

Note: sd: standard deviation. cor: correlation. Inter: Intercept. Cond: Condition. RTs: Response Times. Est.Error: Estimate Error. 95%CrI-low and -up: lower and upper boundary of the 95% Credible Interval. R-hat: R-hat potential scale reduction factor. ESS: Effective Sample Size.

Prior sensitivity analysis

As shown in *Tables S18* and *S19*, refitting the model using priors set 2 (brms' default priors) did not substantially influence our results nor interpretations.

Table S18: *Experiment 3 model results with priors set 2*

	Est.	Est.Err.	95%CrI-low	95%CrI-up	$p_{>0}$	$p_{<0}$	R-hat	ESS
Inter.	.299	.078	.143	.453	1	0	1	9144
Cond.	.181	.091	.002	.358	.976	.024	1	15152
RTs	.757	.265	.236	1.285	.998	.002	1	10118
Cond.:RTs	1.102	.366	.374	1.821	.998	.002	1	12897

Note: Inter: Intercept. Cond.: Condition effect. RTs: Response Times effect. Est.: Estimate. Est.Err.: Estimate Error. 95%CrI-low and -up: lower and upper boundary of the 95% Credible Interval. $p_{>0}$ and $p_{<0}$: proportion of posterior samples greater and lower than zero. R-hat: R-hat potential scale reduction factor. ESS: Effective Sample Size.

Table S19*: Experiment 3 model random effect table with priors set 2*

	Estimate	Est.Error	95%CrI-low	95%CrI-up	R-hat	ESS
sd(Inter.)	.406	.077	.268	.568	1	8326
sd(Cond.)	.254	.139	.015	.536	1	2587
sd(RTs)	1.004	.292	.438	1.594	1	7891
sd(Cond:RTs)	1.541	.460	.569	2.421	1.001	3828
cor(Inter.,Cond.)	-.197	.352	-.766	.590	1	9025
cor(Inter., RTs)	.755	.172	.330	.970	1	9419
cor(Cond., RTs)	-.101	.360	-.762	.626	1	9581
cor(Inter., Cond.:RTs)	-.337	.268	-.807	.224	1	7173
cor(Cond., Cond.:RTs)	.431	.346	-.427	.909	1	2480
cor(RTs, Cond.:RTs)	-.425	.279	-.845	.242	1	6647

Note: sd: standard deviation. cor: correlation. Inter: Intercept. Cond: Condition. RTs: Response Times. Est.Error: Estimate Error. 95%CrI-low and -up: lower and upper boundary of the 95% Credible Interval. R-hat: R-hat potential scale reduction factor. ESS: Effective Sample Size.

Similarly, as shown in *Tables S20* and *S21*, refitting the model using priors set 3 did not substantially influence our results nor interpretations.

Table S20: *Experiment 1 model results with priors set 3*

	Est.	Est.Err.	95%CrI-low	95%CrI-up	$p_{>0}$	$p_{<0}$	BF_{01}	BF_{10}	R-hat	ESS
Inter.	.299	.077	.148	.450	1	0	.020	51.253	1	13064
Cond.	.184	.089	.008	.357	.979	.021	1.435	.697	1	20148
RTs	.736	.244	.254	1.215	.998	.002	.064	15.533	1	16376
Cond.:RTs	1.054	.323	.411	1.682	.999	.001	.028	35.877	1	19474

Note: Inter: Intercept. Cond.: Condition effect. RTs: Response Times effect. Est.: Estimate. Est.Err.: Estimate Error. 95%CrI-low and -up: lower and upper boundary of the 95% Credible Interval. $p_{>0}$ and $p_{<0}$: proportion of posterior samples greater and lower than zero. BF_{01} and BF_{10}: Bayes Factor for the parameter to be equal or different from 0. R-hat: R-hat potential scale reduction factor. ESS: Effective Sample Size.

***Table S21**: Experiment 3 model random effect table with priors set 3*

	Estimate	Est.Error	95%CrI-low	95%CrI-up	R-hat	ESS
sd(Inter.)	.405	.076	.265	.563	1	9520
sd(Cond.)	.269	.139	.020	.539	1.001	3228
sd(RTs)	.877	.290	.295	1.440	1	7464
sd(Cond:RTs)	1.338	.464	.355	2.233	1	4327
cor(Inter.,Cond.)	-.195	.312	-.702	.498	1	10075
cor(Inter., RTs)	.646	.202	.157	.923	1	11017
cor(Cond., RTs)	-.059	.320	-.663	.572	1	15454
cor(Inter., Cond.:RTs)	-.254	.264	-.726	.293	1	13413
cor(Cond., Cond.:RTs)	.361	.308	-.355	.834	1	5526
cor(RTs, Cond.:RTs)	-.314	.290	-.770	.341	1	8190

Note: sd: standard deviation. cor: correlation. Inter: Intercept. Cond: Condition. RTs: Response Times. Est.Error: Estimate Error. 95%CrI-low and -up: lower and upper boundary of the 95% Credible Interval. R-hat: R-hat potential scale reduction factor. ESS: Effective Sample Size.

Experiment 3 – Effect of Heart Rate and Subjective Value on Avoidance Decisions

Chain Convergence has been visually inspected and verified. All chains presented a 'hairy caterpillar' shape. In *Table S22* we provide R-hat and Effective Sample Size (ESS) statistics showing that chains properly mixed.

***Table S22:** Regression table for the logistic model predicting avoidance by condition, RTs, HR and SV*

	Est.	Est.Err.	95%CrI-low	95%CrI-up	p>0	p<0	BF_{01}	BF_{10}	R-hat	ESS
Intercept	.183	.115	-0.043	0.408	.945	.055	6.269	0.160	1	13685
Cond	.322	.150	0.023	0.617	.982	.018	1.871	0.534	1	13306
HR	-.032	.106	-0.239	0.174	.380	.620	22.603	0.044	1	13106
RTs	.298	.104	0.095	0.501	.998	.002	0.454	2.200	1	15587
SV	.229	.240	-0.236	0.706	.829	.171	6.743	0.148	1	14802
Cond:HR	.250	.146	-0.037	0.536	.957	.043	3.888	0.257	1	13505
Cond:RTs	.189	.150	-0.101	0.487	.897	.103	7.793	0.128	1	15914
HR:RTs	-.105	.110	-0.322	0.109	.167	.833	14.226	0.070	1	12732
Cond:SV	-.131	.337	-0.786	0.541	.347	.653	6.732	0.149	1	13446

HR:SV	.171	.251	-0.319	0.664	.754	.246	8.168	0.122	1	12593
RTs:SV	-.291	.236	-0.758	0.165	.108	.892	5.027	0.199	1	13961
Cond:HR:RTs	.177	.156	-0.130	0.485	.873	.127	8.429	0.119	1	13496
Cond:HR:SV	-.946	.354	-1.644	-0.256	.004	.996	0.214	4.681	1	12906
Cond:RTs:SV	.467	.341	-0.203	1.130	.914	.086	3.060	0.327	1	15214
HR:RTs:SV	.515	.268	-0.006	1.051	.973	.027	1.484	0.674	1	12449
Cond:HR:RTs:SV	-.931	.380	-1.685	-0.191	.007	.993	0.309	3.236	1	12963

Note: Cond: condition. HR: maximum instantaneous heart rate difference from baseline. RTs: response times. SV: subjective value. Est.: Estimate. Est.Error: Estimate Error. 95%CrI-low and -up: lower and upper boundary of the 95% Credible Interval. $p_{>0}$ and $p_{<0}$: proportion of posterior samples greater and lower than zero. BF_{01} and BF_{10}: Bayes Factor for the parameter to be equal or different from zero. R-hat: R-hat potential scale reduction factor. ESS: Effective Sample Size.

Experiment 2 and 3 by class

Chain Convergence has been visually inspected and verified. All chains presented a 'hairy caterpillar' shape. In *Tables S23, S24* and *S25* we provide R-hat and Effective Sample Size (ESS) statistics showing that chains properly mixed.

Table S23: *Regression tables for logistic model including classes*

	Est.	Est.Err.	95%CrI-low	95%CrI-up	$p_{>0}$	$p_{<0}$	BF_{01}	BF_{10}	R-hat	ESS
Inter.	.885	.083	.724	1.048	1	0	0	6.541e+15	1	10627
Cond.	.100	.125	-.140	.350	.788	.212	14.705	.068	1	9769
RTs	1.862	.276	1.321	2.413	1	0	0	1.701e+15	1	11107
Class	-.828	.097	-1.020	-.637	0	1	0	2.743e+68	1	10494
Cond:RTs	.227	.425	-.601	1.056	.702	.298	5.095	.196	1	10685
Cond:class	.223	.148	-.073	.511	.931	.069	5.321	.188	1	9352
RTs:class	-1.882	.318	-2.504	-1.263	0	1	0	2.486e+21	1	11310
Cond:RT:class	1.520	.499	.533	2.498	.999	.001	.060	16.635	1	11038

Note: Inter: Intercept. Cond: Condition effect. RTs: response times effect. Class: Effect of the GD class on the SR one. Est.: Estimate. Est.Err.: Estimate Error. 95%CrI-low and -up: lower and upper boundary of the 95% Credible Interval. $p_{>0}$ and $p_{<0}$: proportion of posterior samples greater and lower than zero. BF_{01} and BF_{10}: Bayes Factor for the parameter to be equal or different from zero. R-hat: R-hat potential scale reduction factor. ESS: Effective Sample Size.

Table S24: *Random effect table for logistic model including classes*

	Estimate	Est.Error	95%CrI-low	95%CrI-up	R-hat	ESS

sd(Inter.)	.118	.071	.006	.265	1	4536
sd(Cond.)	.268	.101	.044	.458	1.001	2828
sd(RTs)	.242	.177	.009	.641	1.001	7015
sd(Cond.:RTs)	.628	.331	.037	1.271	1.001	4008
cor(Inter.,Cond.)	-.244	.362	--.800	.556	1	4085
cor(Inter., RTs)	.094	.372	-.648	.748	1	15503
cor(Cond., RTs)	-.012	.364	-.695	.678	1	19263
cor(Inter., Cond.:RTs)	-.096	.360	-.735	.625	1	5883
cor(Cond., Cond.:RTs)	.407	.319	-.369	.868	1.001	1020
cor(RTs, Cond.:RTs)	-.105	.375	-.757	.643	1	10717

Note: Inter.: Intercept. Cond.: Condition effect. RTs: Response times effect. Est.Error: Estimate Error. 95%CrI-low and -up: lower and upper boundary of the 95% Credible Interval. R-hat: R-hat potential scale reduction factor. ESS: Effective Sample Size.

As a robustness check, we fitted separate Bayesian hierarchical logistic models for each class. We found that participants in the GD class exhibited an avoidance rate in the Unpredictable condition not credibly different from random-level (Intercept: 95%CrI=[-.042, .163]; BF_{01}=9.977), but a credibly higher rate in the Predictable condition (Condition effect: β=.326; 95%CrI=[.164, .491]; $p_{<0}$=.0; BF_{10}=60.712; *Figure 4A$_1$* in the main text). Furthermore, while we did not find a credible main effect of RTs in the GD class (95%CrI=[-.301, .341]; BF_{01}=6.134), we did find a credible Condition by RTs interaction (β=1.629; 95%CrI=[1.097, 2.146]; $p_{<0}$=.0; BF_{10}=1.219e+16), showing that in the GD class, slower RTs were associated with a higher probability of avoiding the angry avatar in the Predictable condition, but not in the Unpredictable one (*Figures 4B-C$_1$* in the main text). Conversely, participants in the SR class avoided the angry avatars credibly more than chance throughout the task (Intercept: β=.900; 95%CrI=[.713, 1.099]; $p_{<0}$=.0; BF_{10}=2.421e+15) with no credible difference between conditions (Condition effect: 95%CrI=[-.158, .316], BF_{01}=6.547; *Figure 4A$_2$* in the main text). Moreover, we found a credible main effect of RTs on avoidance rate for this class (RTs: β=1.753; 95%CrI=[1.179, 2.318]; $p_{<0}$=.0; BF_{10}=1.960e+16), with slower RTs associated with a higher probability of avoidance regardless of the condition (Condition x RTs: 95%CrI=[-.497, 1.005], BF_{01}=2.121; *Figure 4B-C$_2$* in the main text and *Table S25* below).

Table S25: *Regression tables for separate logistic models in the GD and SR classes*

	Est.	Est.Err.	95%CrI-low	95%CrI-up	$p_{>0}$	$p_{<0}$	BF_{01}	BF_{10}	R-hat	ESS
GD class										
Inter.	0.059	0.052	-0.042	0.163	0.874	0.126	9.977	1.000e-01	1	17808
Cond.	0.326	0.083	0.164	0.491	1.000	0.000	0.016	6.071e+01	1	21925
RTs	0.020	0.164	-0.301	0.341	0.548	0.452	6.134	1.630e-01	1	22049
Cond.:RTs	1.629	0.267	1.097	2.146	1.000	0.000	0.000	1.219e+16	1	27715
SR class										
Inter.	0.900	0.098	0.713	1.099	1.000	0.000	0.000	2.420e+15	1	17502
Cond.	0.083	0.121	-0.158	0.316	0.757	0.243	6.547	1.530e-01	1	14901
RTs	1.753	0.291	1.179	2.318	1.000	0.000	0.000	1.959e+16	1	16838
Cond.:RTs	0.249	0.385	-0.497	1.005	0.739	0.261	2.121	4.710e-01	1	13549

Note: Inter.: Intercept. Cond.: Condition effect. RTs: Response times effect. Est.: Estimate. Est.Err.: Estimate Error. 95%CrI-low and -up: lower and upper boundary of the 95% Credible Interval. $p_{>0}$ *and* $p_{<0}$*: proportion of posterior samples greater and lower lower than zero.* BF_{01} *and* BF_{10}*: Bayes Factor for the parameter to be equal or different from 0. R-hat: R-hat potential scale reduction factor. ESS: Effective Sample Size.*

Supplementary Methods 4: DDM model fitting and selection

Model fitting

In all experiments Bayesian Hierarchical Drift Diffusion Models were fitted using the Python package *HDDM*[9] (v3.7.13). The package performs parameter estimation using Markov Chain Monte Carlo (MCMC) sampling. For each model three chains were run in parallel with 20,000 iterations each (5,000 of which as burn-in period) and a thinning factor of 3. We used the population-informed priors by Matzke and Wagenmakers[10] to fit the models. In all models the starting point bias was kept fixed and equidistant between boundaries.

Experiment 1

Model Selection

We compared several models by sequentially allowing the drift rate, the boundary separation, the non-decision time, combinations of these or none of them to vary across conditions. In this experiment all criteria favored as best fitting the model allowing the drift rate and the non-decision time to vary across conditions (*Table S26*).

Table S26: *Experiment 1 DDM model selection.*

model	DIC	BPIC
**Cond(a,t0,v)**	**2896.115**	**3055.893**
Cond(a,t0)+v	2925.287	3081.306
Cond(a,v)+t0	2901.317	3066.291
Cond(v+t0)+a	2899.123	3056.410
Cond(a)+t0+v	2931.862	3095.850
Cond(t0)+a+v	2924.362	3077.996
Cond(v)+a+t0	2912.539	3076.557
a+t0+v	2938.374	3101.315

Note: Cond: Condition, between parentheses the parameter(s) allowed to vary. a: Boundary separation. v: Drift rate. t0: Non decision time. DIC: Deviance Information Criterion. BPIC: Bayesian Predictive Information Criterion. Bold: best fitting model.

Model Diagnostic

Chain Convergence has been visually inspected and verified. All chains presented a 'hairy caterpillar' shape. In *Tables S27* and *S28* we provide R-hat and Effective Sample Size (ESS)

statistics showing that chains properly mixed.

Table S27: *Experiment 1 best fitting model group-level mu parameters.*

parameter	Estimate	95%CrI-low	95%CrI-up	$p_{>0}$	$p_{<0}$	R-hat	ESS
a (Intercept)	1.219	1.145	1.298	1	0	1	11142
a (Condition)	.048	.009	.089	.990	.010	1	5022
v (Intercept)	.575	.398	.753	1	0	1	12315
v (Condition)	.344	.226	.460	1	0	1	10385
t0 (Intercept)	.380	.336	.436	1	0	1	12311
t0 (Condition)	.005	-.002	.012	.915	.085	1	3590

Note: a: Boundary Separation. t0: Non decision time. v: Drift rate. Estimate: median posterior estimate. 95%CrI-low and -up: Lower and upper boundaries of the 95% Credible Interval. $p_{>0}$ and $p_{<0}$: Proportion of posterior samples greater and lower than zero. R-hat: R-hat potential scale reduction factor. ESS: Effective Sample Size.

Table S28: *Experiment 1 best fitting model group-level sd parameters*

parameter	Estimate	95%CrI-low	95%CrI-Up	$p_{>0}$	$p_{<0}$	R-hat	ESS
sd (a Intercept)	.274	.225	.342	1	0	1	12121
sd(v Intercept)	.615	.495	.773	1	0	1	14181
sd(t0 Intercept)	.193	.157	.245	1	0	1	13065

Note: a: Boundary separation. t0: Non decision time. v: Drift rate. sd: Standard deviation. Estimate: median posterior estimate. 95%CrI-low and -up: Lower and upper boundaries of the 95% Credible Interval. $p_{>0}$ and $p_{<0}$: Proportion of posterior samples greater and lower than zero. R-hat: R-hat potential scale reduction factor. ESS: Effective Sample Size.

Posterior Predictive Check

In order to assess the reliability of our model we simulated data from the best fitting parameters and we assessed their resemblance to the observed data. Simulated and Observed data present high correlations for avoidance response proportion and median RT for avoidance and approach responses (*Figure S1*).

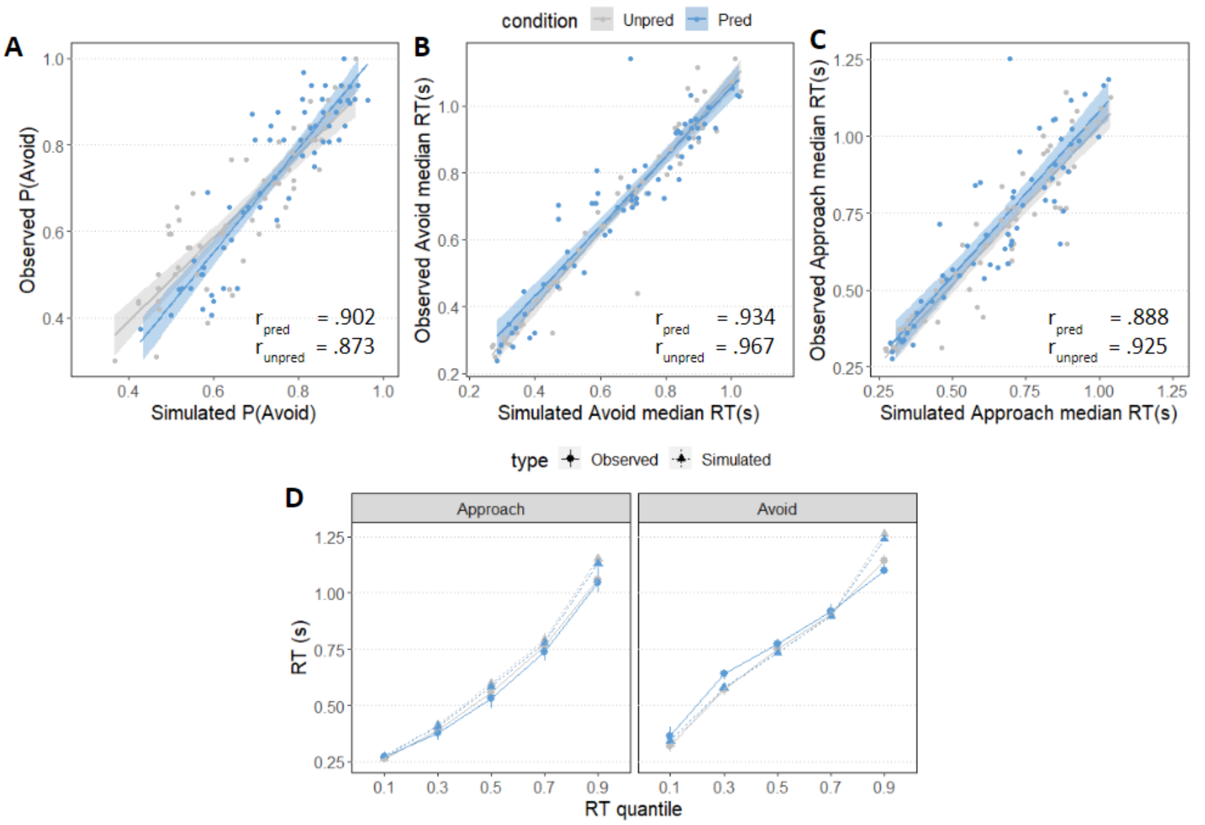

Figure S1: Posterior predictive check for the best fitting DDM in Experiment 1*. Top row: correlation between observed and simulated proportion of avoidance responses, P(Avoid), (A), median RTs for avoidance responses (B), and median RTs for approach responses (C). In all top row panels: points: individual participants, lines: predicted values, shades: 95%CI. (D) Observed and Simulated RTs by quantile of the RTs distribution in the predictable (blue) and unpredictable (grey) condition. In all plots n=60.* Unpred.: Unpredictable condition. Pred.: Predictable condition. RT: Response Time.

Experiment 2

Model Selection

In Experiment 2 all criteria favored as best fitting the model allowing the drift rate and the non-decision time to vary by condition (*Table S29*).

Table S29: *Experiment 2 DDM model selection.*

model	DIC	BPIC
Cond(a,t0,v)	1390.345	1466.925
Cond(a,t0)+v	1401.504	1477.468
Cond(a,v)+t0	1394.436	1473.379
**Cond(v+t0)+a**	**1387.701**	**1462.210**
Cond(a)+t0+v	1405.457	1483.619

Cond(t0)+a+v	1400.267	1476.016
Cond(v)+a+t0	1393.391	1471.016
a+t0+v	1398.445	1470.497

Note: Cond: Condition, between parentheses the parameter(s) allowed to vary. a: Boundary separation. v: Drift rate. t0: Non decision time. DIC: Deviance Information Criterion. BPIC: Bayesian Predictive Information Criterion. Bold: best fitting model.

Model Diagnostic

Chain Convergence has been visually inspected and verified. All chains presented a 'hairy caterpillar' shape. In *Tables S30* and *S31* we provide R-hat and Effective Sample Size (ESS) statistics showing that chains properly mixed.

Table S30: *Experiment 2 best fitting model group-level mu parameters.*

parameter	Estimate	95%CrI-low	95%CrI-up	$p_{>0}$	$p_{<0}$	R-hat	ESS
a	1.150	1.042	1.274	1	0	1	12102
v (Intercept)	.384	.161	.605	1	0	1	11339
v (Condition)	.303	.134	.472	1	0	1	10370
t0 (Intercept)	.370	.304	.461	1	0	1	10790
t0 (Condition)	.007	0	.015	.967	.033	1	2533

Note: a: Boundary Separation. t0: Non decision time. v: Drift rate. Estimate: median posterior estimate. 95%CrI-low and -up: Lower and upper boundary of the 95% Credible Interval. $p_{>0}$ and $p_{<0}$: Proportion of posterior samples greater and lower than zero. R-hat: R-hat potential scale reduction factor. ESS: Effective Sample Size.

Table S31: *Experiment 2 best fitting model group-level sd parameters*

parameter	Estimate	95%CrI-low	95%CrI-Up	$p_{>0}$	$p_{<0}$	R-hat	ESS
sd (a)	.299	.228	.415	1	0	1	12359
sd(t0 Intercept)	.490	.344	.694	1	0	1	11031
sd(v Intercept)	.213	.159	.309	1	0	1	10451

Note: a: Boundary separation. t0: Non decision time. v: Drift rate. sd: Standard deviation. Estimate: median posterior estimate. 95%CrI-low and -up: Lower and upper boundary of the 95% Credible Interval. $p_{>0}$ and $p_{<0}$: Proportion of posterior samples greater and lower than zero. R-hat: R-hat potential scale reduction factor. ESS: Effective Sample Size.

Posterior predictive check

In order to assess the reliability of our model we simulated data from the best fitting

parameters and we assessed their resemblance to the observed data. While the observed and simulated RTs show a high correlation for both avoidance and approach choices and for both the Predictable and Unpredictable condition (*Figure S2B and C*), the correlation of the proportion of avoidance responses between is less good (*Figure S2A*). This is likely due to the facts that: (a) the task contained only a limited number of trials (issue that in Experiment 1 and 3 was balanced by a double number of participants); (b) the sample in Experiment 2 presented latent classes showing different behavioral patterns, as illustrated by finite mixture modeling.

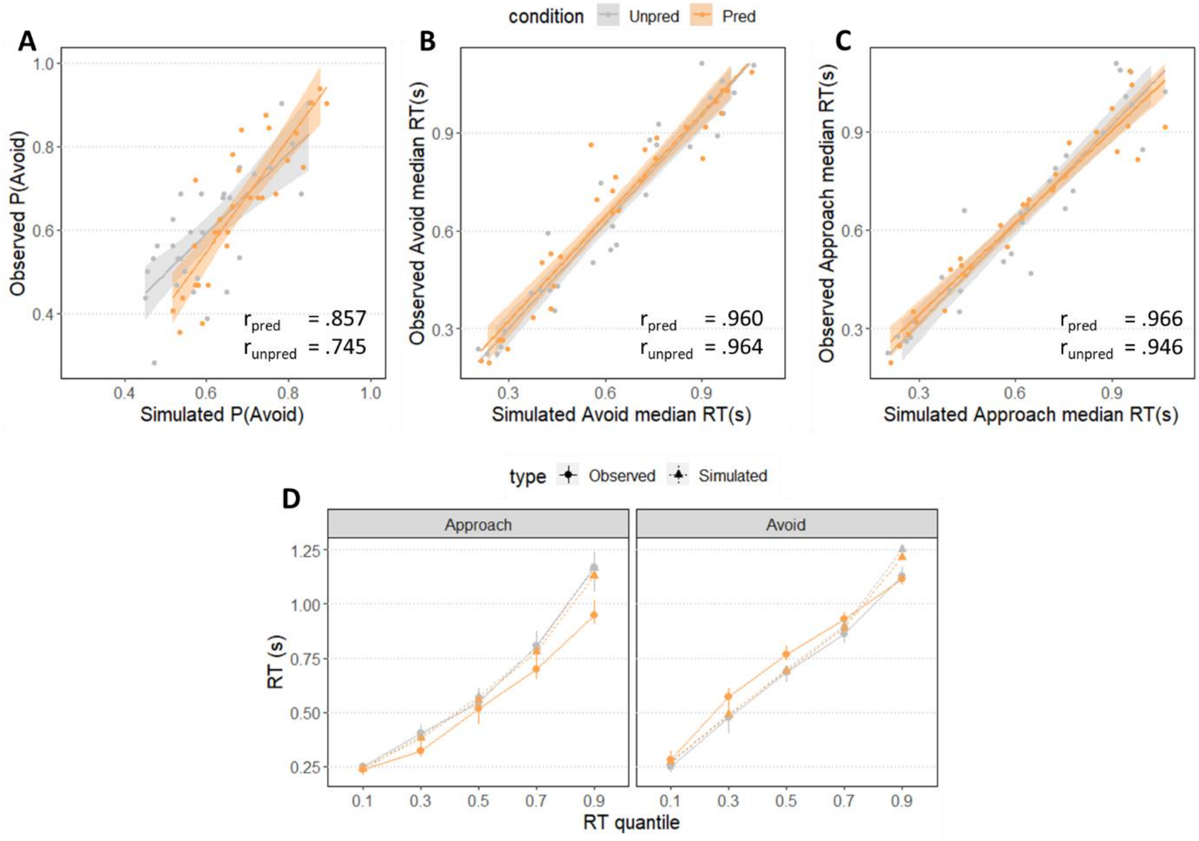

Figure S2: Posterior predictive check for the best fitting DDM in Experiment 2. *Top row: correlation between observed and simulated proportion of avoidance responses, P(Avoid), (A), median RTs for avoidance responses (B), and median RTs for approach responses (C). In all top row panels: points: individual participants, lines: predicted values, shades: 95%CI. (D) Observed and Simulated RTs by quantile of the RTs distribution in the predictable (orange) and unpredictable (grey) condition. In all plots n=30.* Unpred.: Unpredictable condition. Pred.: Predictable condition. RT: Response Time.

Experiment 3

Model Selection

In Experiment 2 all criteria favored as best fitting the model allowing the drift rate and the non-decision time to vary across conditions (*Table S32*).

Table S32: *Experiment 3 DDM model selection.*

model	DIC	BPIC
**Cond(a,t0,v)**	**1744.723**	**1890.045**
Cond(a,t0)+v	1750.789	1895.402
Cond(a,v)+t0	1749.392	1882.588
Cond(v+t0)+a	1745.979	1888.620
Cond(a)+t0+v	1759.447	1896.433
Cond(t0)+a+v	1753.228	1896.395
Cond(v)+a+t0	1755.303	1895.344
a+t0+v	1758.637	1894.979

Note: Cond: Condition, between parentheses the parameter(s) allowed to vary. a: Boundary separation. v: Drift rate. t0: Non decision time. DIC: Deviance Information Criterion. BPIC: Bayesian Predictive Information Criterion. Bold: best fitting model.

Model Diagnostic

Chain Convergence has been visually inspected and verified. All chains presented a 'hairy caterpillar' shape. In *Tables S33* and *S34* we provide R-hat and Effective Sample Size (ESS) statistics showing that chains properly mixed.

Table S33: *Experiment 3 best fitting model group-level mu parameters.*

parameter	Estimate	95%CrI-low	95%CrI-up	$p_{>0}$	$p_{<0}$	R-hat	ESS
a (Intercept)	1.049	.986	1.117	1	0	1	12376
a (Condition)	.035	.002	.068	.981	.019	1	4936
t0 (Intercept)	.340	.297	.393	1	0	1	11841
t0 (Condition)	-.011	-.017	-.006	0	1	1	3349
v (Intercept)	.298	.131	.468	.999	.001	1	9067
v (Condition)	.185	.056	.312	.997	.003	1	9946

Note: a: Boundary Separation. t0: Non decision time. v: Drift rate. Estimate: median posterior estimate. 95%CrI-low and -up: Lower and upper boundaries of the 95% Credible Interval. $p_{>0}$ and $p_{<0}$: Proportion of posterior samples greater and lower than zero. R-hat: R-hat potential scale reduction factor. ESS: Effective Sample Size.

Table S34: *Experiment 2 best fitting model group-level sd parameters*

parameter	Estimate	95%CrI-low	95%CrI-Up	$p_{>0}$	$p_{<0}$	R-hat	ESS
sd (a Intercept)	.240	.196	.299	1	0	1	11313
sd(t0 Intercept)	.182	.147	.234	1	0	1	12479
sd(v Intercept)	.543	.432	.687	1	0	1	11297

Note: a: Boundary separation. t0: Non decision time. v: Drift rate. sd: Standard deviation. Estimate: median posterior estimate. 95%CrI-low and -up: Lower and upper boundary of the 95% Credible Interval. $p_{>0}$ *and* $p_{<0}$*: Proportion of posterior samples greater and lower than zero. R-hat: R-hat potential scale reduction factor. ESS: Effective Sample Size.*

Posterior predictive check

In order to assess the reliability of our model we simulated data from the best fitting parameters and we assessed their resemblance to the observed data. Simulated and Observed data present high correlations for avoidance response proportion and median RT for avoidance and approach responses (*Figure S3A-C*).

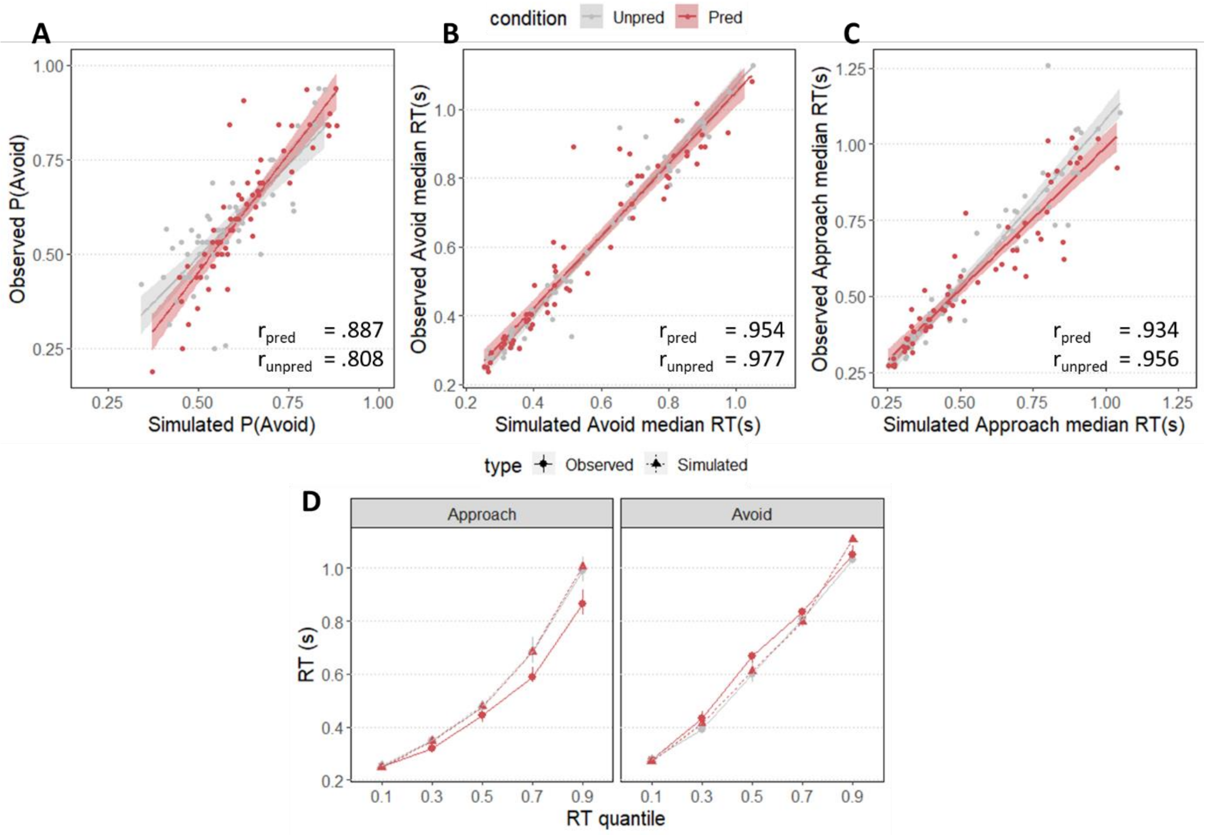

Figure S3: Posterior predictive check for the best fitting DDM in Experiment 3. *Top row: correlation between observed and simulated proportion of avoidance responses, P(Avoid), (A), median RTs for avoidance responses*

(B), and median RTs for approach responses (C). In all top row panels: points: individual participants, lines: predicted values, shades: 95%CI. (D) Observed and Simulated RTs by quantile of the RTs distribution in the predictable (red) and unpredictable (grey) condition. In all plots n=60. Unpred.: Unpredictable condition. Pred.: Predictable condition. RT: Response Time.

Experiment 2 and 3 by class

Model Selection

In Experiment 2 all criteria favored as best fitting the model allowing the drift rate and the non-decision time to vary across conditions (*Table S35*).

Table S35: *DDM model selection including latent classes*

model	DIC	BPIC
Class x Cond(a,t0,v)	2831.372	3211.618
Class x Cond(a,t0)+v	2934.104	3266.796
Class x Cond(a,v)+t0	3075.491	3387.706
**Class x Cond(v+t0)+a**	**2781.559**	**3082.148**
Class x Cond(a)+t0+v	3178.824	3445.030
Class x Cond(t0)+a+v	3150.556	3370.532
Class x Cond(v)+a+t0	3058.893	3296.496
Class(a+t0+v)	3149.917	3351.464

Note: Cond: Condition, between parentheses the parameter(s) allowed to vary. a: Boundary separation. v: Drift rate. t0: Non decision time. DIC: Deviance Information Criterion. BPIC: Bayesian Predictive Information Criterion. Bold: best fitting model.

Model Diagnostic

Chain Convergence has been visually inspected and verified. All chains presented a 'hairy caterpillar' shape. In *Table S36* and *S37* we provide R-hat and Effective Sample Size (ESS) statistics showing that chains properly mixed.

Table S36: *Best fitting model group-level mu parameters.*

parameter	Estimate	95%CrI-low	95%CrI-up	$p_{>0}$	$p_{<0}$	R-hat	ESS
GD class: a	1.163	1.067	1.261	1	0	1	12913
SR class: a	1.053	.988	1.121	1	0	1	13626
GD class: v Unpred.	.898	.735	1.068	1	0	1	8196
SR class: v Unpred.	.061	-.059	.178	.839	.161	1	9234

GD class: v Pred.	1.109	.850	1.370	1	0	1	13902
SR class: v Pred.	.303	.123	.476	1	0	1	13924
GD class: t0 Unpred.	.380	.323	.440	1	0	1	13960
SR class: t0 Unpred.	.325	.286	.371	1	0	1	13293
GD class: t0 Pred.	.410	.348	.475	1	0	1	13887
SR class: t0 Pred.	.330	.288	.375	1	0	1	13369

Note: a: Boundary Separation. t0: Non decision time. v: Drift rate. Unpred. and Pred.: Unpredictable and Predictable conditions. Estimate: median posterior estimate. 95%CrI-low and -up: Lower and upper boundaries of the 95% Credible Interval. $p_{>0}$ and $p_{<0}$: Proportion of posterior samples greater and lower than zero. R-hat: R-hat potential scale reduction factor. ESS: Effective Sample Size.

Table S37: *Best fitting model group-level SD parameters*

parameter	Estimate	95%CrI-low	95%CrI-Up	$p_{>0}$	$p_{<0}$	R-hat	ESS
sd (a)	.255	.217	.304	1	0	1	13007
sd (v Unpred.)	.292	.172	.414	1	0	1.001	2027
sd(v Pred.)	.589	.473	.725	1	0	1	9579
sd (t0 Unpred.)	.181	.152	.219	1	0	1	12766
sd(t0 Pred.)	.188	.159	.228	1	0	1	12257

Note: a: Boundary separation. t0: Non decision time. v: Drift rate. Unpred. and Pred.: Unpredictable and Predictable conditions. sd: Standard deviation. Estimate: median posterior estimate. 95%CrI-low and -up: Lower and upper boundary of the 95% Credible Interval. $p_{>0}$ and $p_{<0}$: Proportion of posterior samples greater and lower than zero. R-hat: R-hat potential scale reduction factor. ESS: Effective Sample Size.

Posterior predictive check

To assess the reliability of our model, we simulated data from the best fitting parameters and we assessed their resemblance to the observed data. Simulated and Observed data presented high correlations for avoidance response proportion and median RT for avoidance and approach responses (*Figure S4*).

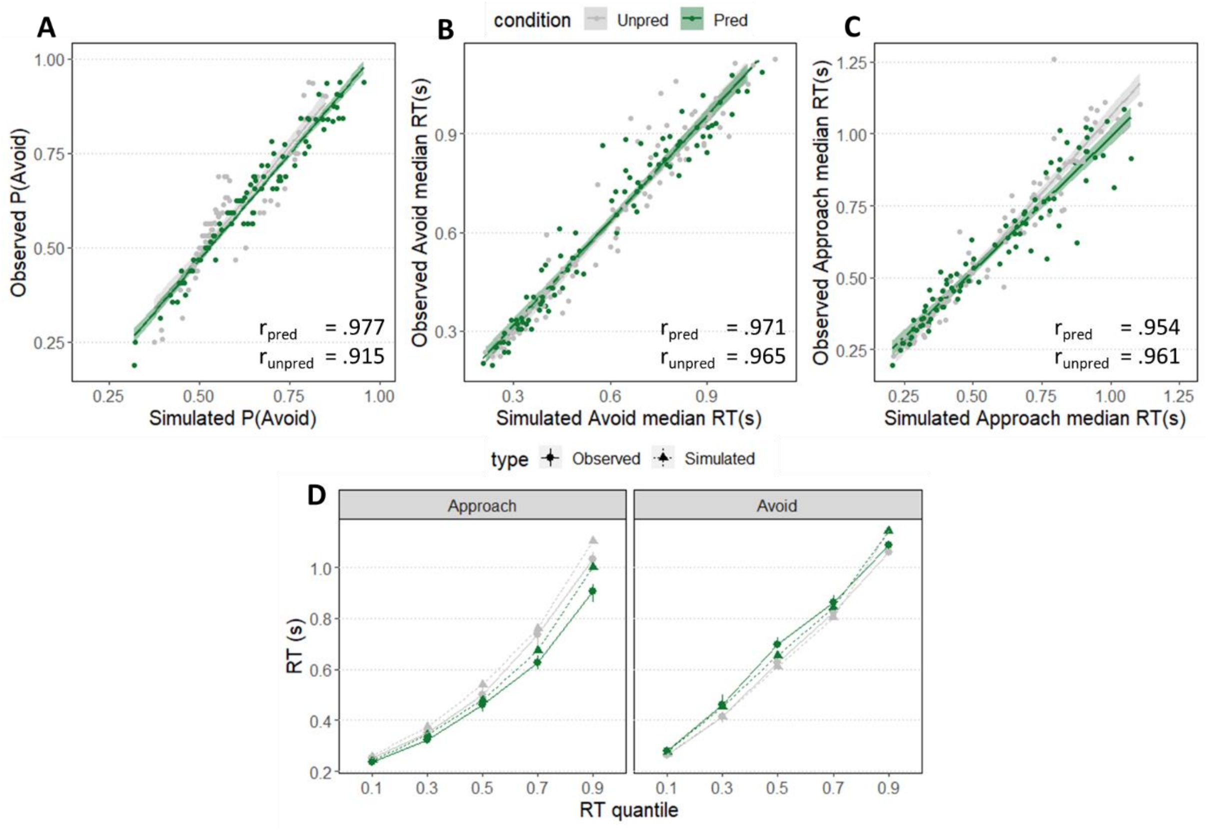

Figure S4: Posterior predictive check for the best fitting DDM including latent classes. *Top row: correlation between observed and simulated proportion of avoidance responses, P(Avoid), (A), median RTs for avoidance responses (B), and median RTs for approach responses (C). In all top row panels: points: individual participants, lines: predicted values, shades: 95%CI. (D) Observed and Simulated RTs by quantile of the RTs distribution in the predictable (green) and unpredictable (grey) condition. In all plots n=90.* Unpred.: Unpredictable condition. Pred.: Predictable condition. RT: Response Time.

Supplementary Note 1: Pilot Experiment

Materials and Methods

Participants

Thirty-three volunteers participated in this pilot. Three participants have been excluded from the analyses because they had to interrupt the experiment due cybersickness symptoms, as well as seven participants who did not interrupt the experiment but still have reported high cybersickness symptoms. This left us with a sample of 23 participants (15 females; mean age ± SD = 24.91 ± 5.07). Participants were left- (17%) or right-handed (83%), and they had no history of neurological or psychiatric disorder (see *Supplementary Methods 2* for a full list of inclusion criteria). The experimental protocol was approved by INSERM and licensed by the local research ethics committee (IRB00003888 – Avis 18-544-ter –25.10.2021). Participants provided informed written consent and were compensated for their participation.

Stimuli

The stimuli were identical to Experiments 1, 2 and 3.

Task

The task design used for this pilot was almost identical to the one described in the *Experiment 1* section, except for: (a) Early choices (i.e. choices before the doors' opening) were not followed by the text prompt saying: "*Ne pas choisir avant l'ouverture des portes!*" ("*Do not choose before the doors opening!*") and the trial was not restarted; (b) trials ended with participants' view rotating toward the elevators' door to face the avatar. This rotation was removed from the final version of the task as it caused cybersickness symptoms.

Equipment

The VR HMD and controllers were the same as in the other experiments. Differently from main experiments, the software of this pilot ran on a Razer Blade 17 laptop with 2.3GHz 8-core Intel i7-11800H processor, a 32GB DDR4 3200 MHz RAM, and a NVIDIA GeForce RTX 3070 8GB GDDR6 graphic card.

Data Analysis

The analyses were identical to Experiments 1 to 3. Eleven trials were excluded due to a response time lower than 150 ms (0.80%).

Results

The best fitting model has been found to be the one including the Condition, the RTs (grand average centered) and their interaction as predictors. Participants avoided the angry avatar credibly more in the Predictable condition than in the Unpredictable one (Condition effect: β=.429; 95%CrI=[.141, .707]; $p_{<0}$=.002; BF_{10}=4.401) while avoidance probability was not credibly different than chance level in the Unpredictable condition (Intercept: 95%CrI=[-.054, .479]; BF_{01}=5.716). Both the main effect of RTs and their interaction effect with Condition were found to be credible (RTs effect: β=.728; 95%CrI=[.011, 1.452]; $p_{<0}$=.024; BF_{10}=1.087; Condition x RTs interaction: β=1.108; 95%CrI=[.078, 2.107]; $p_{<0}$=.017; BF_{10}=1.897; *Supplementary Table S38* and *Supplementary Figure S5*).

Table S38: *Pilot experiment regression table*

	Est.	Est.Err.	95%CrI-low	)5%CrI-Up	$p_{>0}$	$p_{<0}$	BF_{01}	BF_{10}	R-hat	ESS
Inter.	.208	.135	-.054	.479	.939	.061	5.716	.175	1.001	10593
Cond.	.429	.141	.148	.707	.998	.002	.227	4.401	1	18733
RTs	.728	.366	.011	.452	.976	.024	.920	1.087	1	17141
Cond.:RTs	1.108	.518	.078	2.107	.983	.017	.527	1.897	1	16738

Note: Inter: Intercept. Cond.: Condition effect. RTs: Response Times effect. Est.: Estimate. Est.Error: Estimate Error. 95%CrI-low and -up: lower and upper boundary of the 95% Credible Interval. p>0 and p<0: proportion of posterior samples greater and lower than zero. BF_{01} *and* BF_{10}*: Bayes Factor for the parameter to be equal or different from zero. Rhat: R-hat potential scale reduction factor. ESS: Effective Sample Size.*

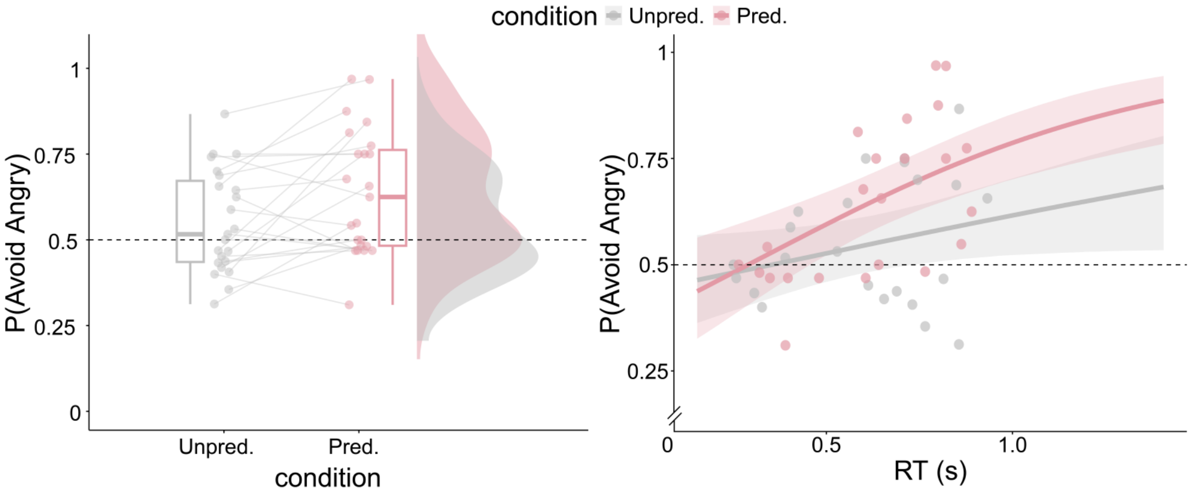

Figure S5: main results from the pilot experiment. *(left) Proportion of avoidance choices across conditions. Boxplots represent first, second (median) and third quartile. Whiskers are drawn within the 1.5 interquartile range. (right) Predicted probability of avoidance choices as a function of RTs. Points: observed avoidance rates over median RTs for each participant. Solid lines: group level predicted values. Shaded areas: 95% credible intervals. Dashed lines: chance level (50%). In both plots n=23. Gray: Unpredictable condition. Pink: Predictable condition. Unpred.: Unpredictable condition. Pred.: Predictable condition. RT: Response Time.*

Supplementary Note 2: Analyses on response times

Response Times were modeled trial-by-trial using a Bayesian GLMM with a shifted log-normal likelihood function. We considered the Condition (treatment coded: Unpredictable = 0, Predictable = 1), the Response (treatment coded: Approach angry = 0, Avoid angry = 1) and their interaction as predictors. On a separate approach, we conducted a similar model comparison on a dataset including trials in which no angry face was present at the moment of choice (Neutral trials). In this case, we considered the Condition, the presence of Threat in the trial (treatment coded: Neutral = 0, Threat = 1) and their interaction as predictors. Since these models were ran on the pooled dataset of experiments 2 and 3 (overall N=90, n=47 females, n=43 males; mean age ± SD = 23.14 ± 4.2), we also added as a predictor the class of the participant (SR class = 0, GD class = 1). Where necessary, post-hoc analyses have been conducted using the R package *emmeans*[13] (v1.7.0).

When focusing on Threat trials, model comparison revealed the best fitting model to be the one including the effect of Condition, Response, GD class and interactions. We found a credible class by Response by Condition three-way interaction (β=.097; 95%CrI=[.021, .993]; $p_{<0}$=.007; BF_{10}=2.317). Post-hoc analyses revealed that the SR class presented credibly slower RTs compared to the GD class only for avoidance responses in the Unpredictable condition (emm=.227, 95%CrI=[.035, .413]) but not in the Predictable condition (95%CrI=[-.021, .359]), with no differences for the approach responses between classes (Predictable: 95%CrI=[-.025, .340], Unpredictable: 95%CrI=[-.070, .303]).

Finally, when fitting models on RTs between Neutral and Threat trials, we found the best fitting model was the one including the main effect of Condition, Threat and Class, and their interaction. However, we only found a credible effect of Class (β=-.195; 95%CrI=[-.385, -.002]; $p_{>0}$=24; BF_{10}=2.261), showing that overall, the GD class presented slower RTs compared to the SR class.

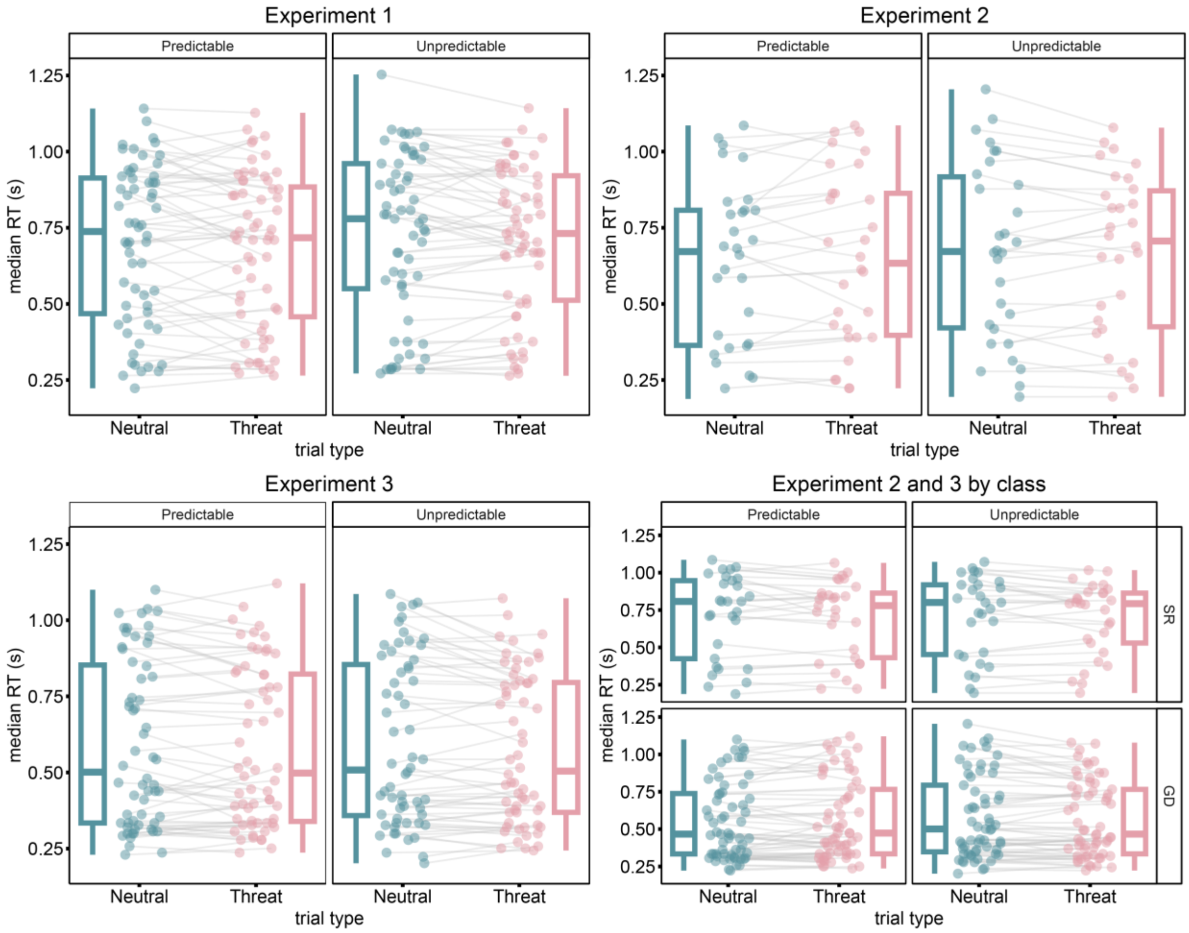

Figure S6: Effect of threat on RTs. *Median response times by Condition (Unpredictable vs Predictable) and trial type (Neutral vs Threat) for each experiment and by latent Classes (SR: Stimulus-Response, GD: Goal-Directed class). Points represent individual participants. Boxplots represent first, second (median) and third quartile. Whiskers are drawn within the 1.5 interquartile range. Experiment 1: n=60, Experiment 2: n=30, Experiment 3: n=60, SR class: n=31, GD class: n=69. Teal: Neutral trials. Pink: Threat trials.*

Supplementary Note 3: Effect of individual dyads

To control for the effect of each dyad of avatars on the main results, as a robustness check we re-fitted the same models predicting the choices presented in the main text, but including the specific avatar dyad presented in each trial as a random effect. Therefore, in R syntax, such models had the form: response ~ 1 + condition * RT + (1 + condition * RTc| ID) + (1 | avatar dyad). As shown in the following tables (*Tables S39 – S42*), this additional complexity did not affect the results presented in the manuscript, showing generalizability beyond the specific dyad of avatars used.

Table S39: *Experiment 1 (n=60) GLMM parameters estimate with (1 | avatar dyad)*

	Est.	Est.Err	95%CrI-low	95%CrI-up	$p_{>0}$	$p_{<0}$	BF_{01}	BF_{10}	R-hat	ESS
Inter.	.710	.105	.505	.919	1	0	0	6.781e+14	1	13085
Cond.	.468	.114	.249	.695	1	0	.003	301.315	1	18959
RTs	1.089	.248	.607	1.582	1	0	.002	566.578	1	17399
Cond.:RTs	.304	.371	-.435	1.019	.796	.204	4.586	.218	1	15331

Table S40: *Experiment 2 (n=30) GLMM parameters estimate with (1 | avatar dyad)*

	Est.	Est.Err	95%CrI-low	95%CrI-up	$p_{>0}$	$p_{<0}$	BF_{01}	BF_{10}	R-hat	ESS
Inter.	.420	.116	.193	.650	1	0	.035	28.571	1	12968
Cond.	.305	.123	.064	.544	.992	.008	.956	1.036	1	19632
RTs	.434	.304	-.146	1.040	.928	.072	3.131	.319	1	15862
Cond.:RTs	1.492	.378	.724	2.215	1	0	.007	149.010	1	18828

Table S41: *Experiment 3 (n=60) GLMM parameters estimate with (1 | avatar dyad)*

	Est.	Est.Err	95%CrI-low	95%CrI-up	$p_{>0}$	$p_{<0}$	BF_{01}	BF_{10}	R-hat	ESS
Inter.	.303	.085	.137	.472	1	0	.076	13.100	1	11433
Cond.	.189	.089	.016	.364	.983	.017	2.977	.336	1	17034
RTs	.741	.248	.248	1.227	.998	.002	.125	7.968	1	13244
Cond.:RTs	1.124	.336	.459	1.780	.999	.001	.032	31.263	1	15424

Table S42: *Classes (n=90) GLMM parameters estimate with (1 | avatar dyad)*

	Est.	Est.Err	95%CrI-low	95%CrI-up	$p_{>0}$	$p_{<0}$	BF_{01}	BF_{10}	R-hat	ESS
Inter.	.894	.091	.716	1.077	1	0	0	2.714e+15	1	12693
Cond.	.101	.125	-.148	.350	.790	.210	14.588	.069	1	13610
RTs	1.904	.278	1.369	2.454	1	0	0	1.014e+15	1	15025
class	-.837	.097	-1.033	-.647	0	1	0	8.157e+60	1	14635

Cond.:RTs	.201	.430	-.640	1.041	.680	.320	5.249	.190	1	14913
Cond.:Class	.227	.149	-.067	.515	.936	.064	5.350	.187	1	13604
RTs:Class	-1.925	.325	-2.569	-1.293	0	1	0	2.446e+16	1	15331
Cond.:RTs:Class	1.572	.503	.580	2.563	.999	.001	.052	19.406	1	14708

Note for tables S43-S46: Inter. Intercept. Cond: condition effect. RTs: response times effect. Est.: Estimate. Est.Err.: Estimate Error. 95%CrI-low and -up: lower and upper boundary of the 95% Credible Interval. $p_{>0}$ and $p_{<0}$: proportion of posterior samples greater and lower than zero. BF_{01} and BF_{10}: Bayes Factor for the parameter to be equal or different from 0. R-hat: R-hat potential scale reduction factor. ESS: Effective Sample Size.

Supplementary Note 4: Analyses on the effect of avatars' sex

We further tested if the sex of the avatars in the virtual environment had an effect on participants' avoidance behavior. To do so, we ran analyses on the pooled experiments of experiment 2 and 3 (overall N=90, n=47 females, n=43 males; mean age ± SD = 23.14 ± 4.2). We fitted the same logistic model on avoidance choice that resulted to be the best fitting (response ~ condition * RT; see main text), but we added the sex of the avatar as a 2-level categorical predictor (treatment coded: female avatar = 0, male avatar = 1) and we considered its interactions with other effect in the model.

A credible condition by RT by avatar sex interaction (β=-1.020; 95%CrI=[-1.819, -.233]; $p_{>0}$=.006; BF_{10}=3.804; *Table S43* and *Figure S7*) was found, showing that with female avatars the difference in avoidance rate between conditions increased with slower RTs (coherently with the results presented in the main text), whereas this effect was reduced with male avatars. One possible interpretation of this result is that male avatars are perceived as more threatening, thereby fostering SR avoidance strategy even at slower RTs, hence the reduced difference between conditions. To test this possibility, we assessed whether ratings on the Subjective Evaluation Task differed between male and female avatars by running a 2 (emotion: Neutral vs. Angry) by 2 (sex: Female vs. Male) within-subjects ANOVA. We found that participants reported significantly greater value for approaching neutral avatars than angry avatars (emotion effect: $F_{(1,28)}$=517, p<.001, η_g^2=.946), whereas both the effect of avatar's sex ($F_{(1,28)}$=.111,p=.741, η_g^2=.004) and the emotion by avatar's sex interaction ($F_{(1,28)}$=.4.011,p=.055, η_g^2=.125) were not significant. Therefore, the interpretation of an increased threat value for male avatars must be taken with caution.

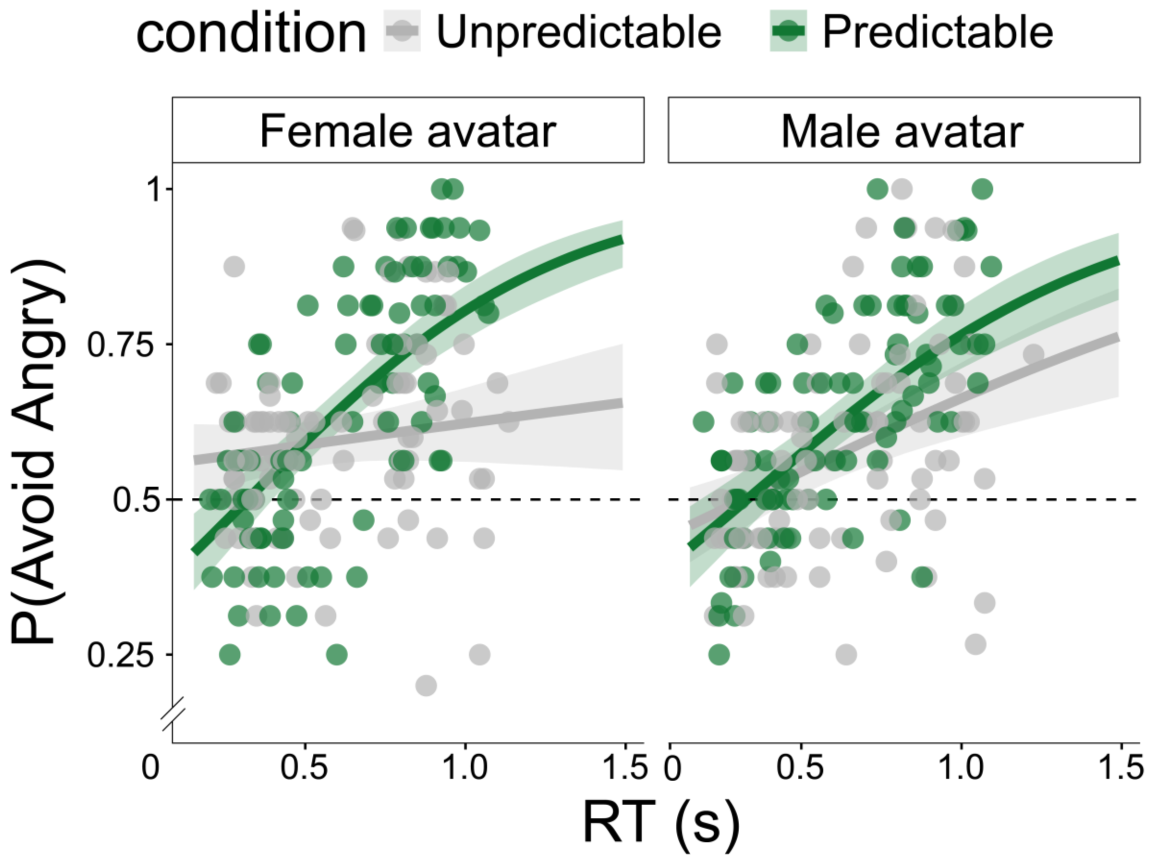

Figure S7: Condition by RTs by avatar's sex interaction on p(avoid). *Predicted probability of avoidance choices, P(Avoid Angry), as a function of RTs (solid lines) and by condition and avatar's sex. Points: observed avoidance rates over median RTs for each participant. Shaded areas: 95% credible intervals. N=90.* RT: Response Time.

Table S43: *Model results and diagnostic for each parameter of the response ~ condition * RT * avatar sex model*

	Est.	Est.Err	95%CrI-low	95%CrI-Up	$p_{>0}$	$p_{<0}$	BF_{01}	BF_{10}	R-hat	ESS
Inter.	390	078	238	542	.	)	)	..737e+17	.	.5093
Cond.	237	095	051	423	992	008	..281	781	.	.6310
RTs	293	238	.170	764	891	109	.048	198	.	.5913
sex	.090	082	.252	072	138	863	.7.632	057	.	2017
Cond.:RTs	..785	323	..153	.420	.	)	)	-.266e+15	.	.6533
Cond.:sex	.024	119	.258	209	209	578	.9.827	005	.	1045
RTs:sex	705	275	164	..251	994	006	311	.213	.	.9874
Cond.:RTs:sex	1.020	404	1.819	.233	006	994	263	.804	.	0884

Note: Inter.: Intercept. Cond.: Condition effect. RTs: response times. sex: Effect of male avatars compared to female avatars. Est.: Estimate. Est.Err.: Estimate Error. 95%CrI-low and -up: lower and upper boundary of the 95% Credible Interval. $p_{>0}$ *and* $p_{<0}$*: proportion of posterior samples greater and lower than zero.* BF_{01} *and* BF_{10}*: Bayes Factor for the parameter to be equal or different from 0. R-hat: R-hat potential scale reduction factor. ESS: Effective Sample Size.*

We then tested whether this effect was moderated by the participants' own sex. To do this, we fitted the same model but added the participants' sex as a predictor (treatment coded: Female = 0, Male = 1) and its interaction with other regressors. We found that this model did not fit the data better than the simple version (ΔWAIC = 9.73), showing generalizability across participants' sexes.

Supplementary Note 5: Avoidance rate across experimental blocks

To test for possible learning effects in the task and study the evolution of avoidance behavior across experimental blocks, we ran a model predicting avoidance decisions including as predictors the block (First vs. Last), the condition, the class, the RTs and their interactions, focusing on the pooled sample of experiments 2 and 3 (overall N=90, n=47 females, n=43 males; mean age ± SD = 23.14 ± 4.2). We kept a maximal random structure for the within-subjects' effects.

We found a marginally significant Condition by Block by RTs by Class 4-way interaction (p=.054, see *Table S39*). To better understand the interaction, we ran the same model separately for each class (*Table S40*), and found that the interaction between block and condition was significant only for the GD class (SR: β=-.074, 95%CrI=[-.693, .554], $p_{>0}$=.408, BF_{01}=7.561; GD: β=.397, 95%CrI=[.007, .788], $p_{<0}$=.023, BF_{10}=.567), suggesting that in the GD class, but not in the SR, avoidance rates increased from the first to the last block only for the predictable, but not for the unpredictable, condition (see *Figure 4D*, main manuscript, and *Table S41)*. This indicates the GD strategy builds up over time as participants learn that they can only predict the outcome of their action in the predictable condition. Consistently, this is not the case for the group of participants whose avoidance is SR-driven.

Table S44*: Regression table for the p(avoid) ~ block * condition * class * RTs model*

	Est.	Est.Err	95%CrI-low	95%CrI-up	$p_{>0}$	$p_{<0}$	BF_{01}	BF_{10}	R-hat	ESS
Inter	.679	.139	.408	.954	1	0	0	7.278e+13	1	10006
Conc	.105	.205	-.296	.510	.699	.301	10.569	.095	1	9662
Bloc	.397	.235	-.058	.868	.956	.044	2.478	.404	1.001	9552
RT	1.335	.422	.523	2.171	1	0	.028	36.330	1	11358
Clas	-.613	.163	-.931	-.296	0	1	.005	207.391	1	9918
Cond:BLoc	-.076	.311	-.685	.533	.404	.596	7.620	.131	1	9283
Cond:RT	.532	.620	-.694	1.754	.807	.193	2.857	.350	1	11298
Block:RT	1.286	.719	-.106	2.696	.963	.037	.752	1.330	1	11919
Cond:Clas	.022	.242	-.456	.494	.541	.459	10.154	.098	1	9662
Block:Clas	-.371	.273	-.912	.169	.086	.914	3.797	.263	1	9726
RT:Clas	-1.482	.485	-2.439	-.540	.001	.999	0.042	23.925	1	11418
Cond:Block:RT	-1.487	.927	-3.310	.334	.055	.945	.736	1.359	1	11991
Cond:Block:Clas	.464	.367	-.251	1.181	.898	.102	3.070	.326	1	9458
Cond:RTs:CLas	.777	.721	-.629	2.192	.859	.141	1.953	.512	1	11827

Block:RTs:Clas	-.613	.815	-2.214	.967	.224	.776	2.353	.425	1	11619
l:Block:RTs:Clas	2.046	1.064	-.039	4.151	.973	.027	.381	2.627	1	12376

Note: Inter: Intercept. Cond: Effect of the Predictable condition on the Unpredictable condition. Block: Effect of the last block on the first one. RT: Response Time. Class: effect of the GD class on the SR class. Est.: Estimate. Est.Err.: Estimate Error. 95%CrI-low and –up: 95% Credible Interval, lower and upper boundary. $P_{>0}$ and $p_{<0}$: proportion of posterior samples greater and lower than zero. BF_{01} and BF_{10}: Bayes Factor for the null (the parameter is not different from zero) and alternative (the parameter is different from zero) hypothesis. R-hat: potential scale reduction factor. ESS: Effective Sample Size.

Table S45*: Regression table for the p(avoid) ~ block * condition * RTs model for each class*

	Est.	Est.Err	95%CrI-low	95%CrI-up	$p_{>0}$	$p_{<0}$	BF_{01}	BF_{10}	R-hat	ESS
				SR Class						
Inte	.682	.144	.405	.967	1	0	0	3.103e+07	1	12302
Con	.110	.207	-.294	.517	.701	.299	10.316	.097	1	11618
Bloc	.411	.247	-.061	.908	.956	.044	2.499	.400	1	11342
RT	1.336	.443	.476	2.216	.999	.001	.061	16.498	1	14096
Cond:Bloc	-.074	.319	-.693	.554	.408	.592	7.561	.132	1	11526
Cond:RT	.545	.656	-.741	1.845	.795	.205	2.893	.346	1	13734
Block:RT	1.441	.774	-.074	2.957	.969	.031	.614	1.629	1	13850
Cond:Block:RT	-1.751	1.012	-3.725	.239	.042	.958	.558	1.791	1	13636
				GD Class						
Inte	.066	.086	-.103	.231	.780	.220	21.539	.046	1	15650
Con	.127	.131	-.129	.386	.833	.167	12.459	.080	1	15462
Bloc	.021	.143	-.261	.302	.556	.444	17.274	.058	1	13570
RT	-.126	.270	-.655	.402	.320	.680	8.793	.114	1	15532
Cond:Bloc	.397	.200	.007	.788	.977	.023	1.763	.567	1	14762
Cond:RT	1.261	.427	.429	2.093	.999	.001	.073	13.711	1	15040
Block:RT	.616	.451	-.264	1.505	.914	.086	2.159	.463	1	13534
Cond:Block:RT	.685	.654	-.587	1.953	.853	.147	2.279	.439	1	15178

Note: Inter: Intercept. Cond: Effect of the Predictable condition on the Unpredictable condition. Block: Effect of the last block on the first one. RTs: Response Times. Class: effect of the GD class on the SR class. Est.: Estimate. Est.Err.: Estimate Error. 95%CrI-low and –up: 95% Credible Interval, lower and upper boundary. $P_{>0}$ and $p_{<0}$: proportion of posterior samples greater and lower than zero. BF_{01} and BF_{10}: Bayes Factor for the null (the parameter is not different from zero) and alternative (the parameter is different from zero) hypothesis. R-hat: potential scale reduction factor. ESS: Effective Sample Size.

Table S46: *Proportion of avoidance responses by class, condition and block.*

	SR class		GD class	
Block	Predictable	Unpredictable	Predictable	Unpredictable
first	.697	.680	.533	.521
last	.759	.774	.599	.523

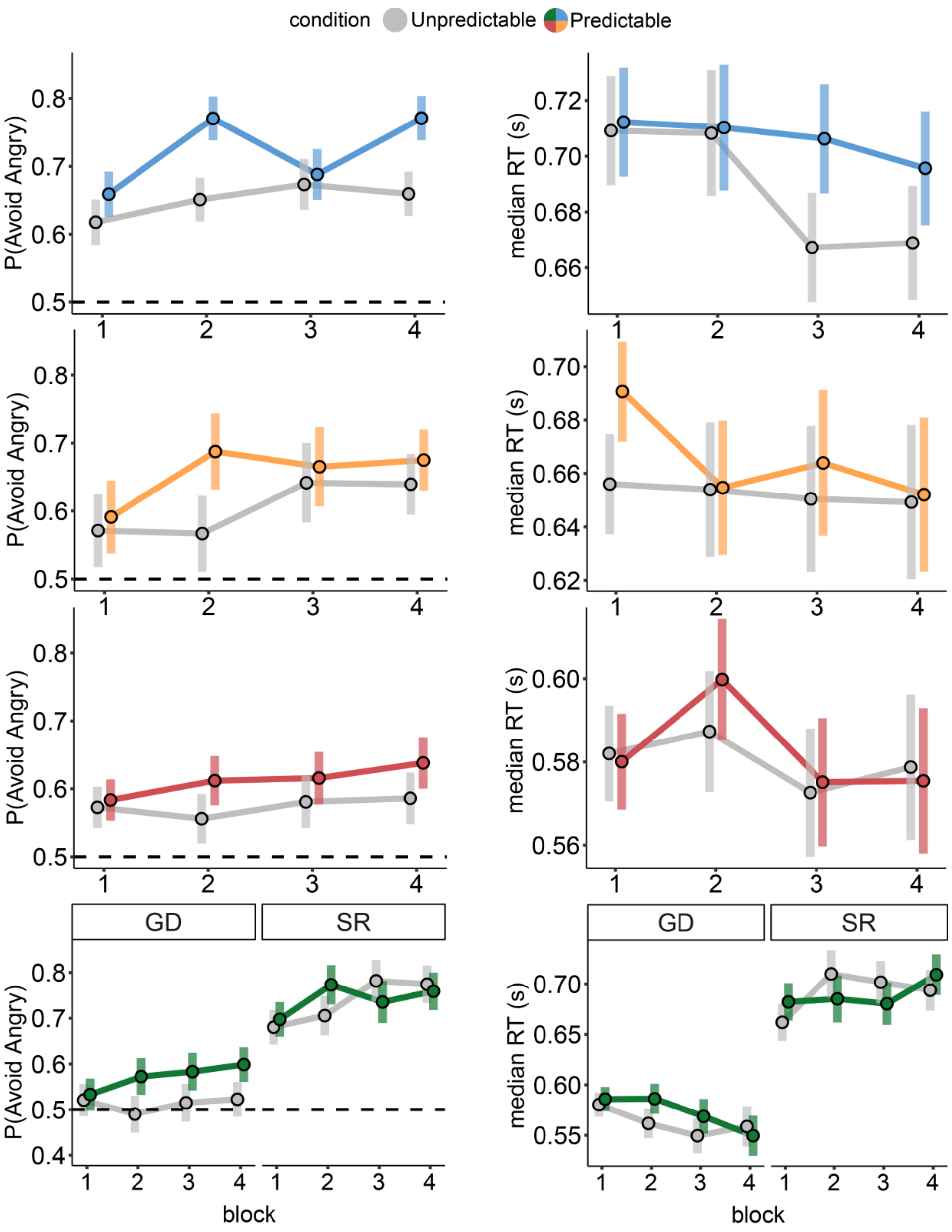

Figure S8: Effect of experimental block. *Proportion of avoidance responses, P(Avoid Angry), (left) and median response times (right) by Condition (Unpredictable in vs Predictable) for each experiment and by class (Experiment 2 and 3 combined). Dots: mean proportion of responses or median response time over the whole sample. Vertical lines: within subjects 95% confidence interval. Top row: experiment 1, n=60. Second row: experiment 2, n=30. Third row: experiment 3, n=60. Bottom row: analyses by class, GD class: n=61, SR Class: n=31. Gray: Unpredictable condition: Blue, orange, red and green: Predictable condition in Experiment 1, 2, 3 and*

by in analyses by class.

Supplementary Note 6: Test on GLMMs standardized coefficients

In the main text, we fitted logistic mixed effect models predicting avoidance choices by condition and response times. Since the Condition variable was treatment coded (Unpredictable = 0, Predictable = 1), the intercept of this model represents the probability of avoidance in the Unpredictable condition, while the Condition effect represents the log-odds change in the probability in avoidance probability between the two conditions. The coefficient for the Intercept can therefore be interpreted as a signature of SR processes (i.e., non-instrumental avoidance when the outcome is not predictable), whereas the condition effect coefficient could be interpreted as a signature of GD processes. Thus, by comparing the standardized coefficient of intercept and condition, the relative contribution of GD and SR processes to participants' avoidance behavior can be tested.

We focused this exploratory analysis on the pooled sample of experiments 2 and 3 (overall N=90, n=47 females, n=43 males; mean age ± SD = 23.14 ± 4.2). We performed a 2 (coefficient: Intercept vs. Condition effect) by 2 (latent class: SR vs. GD) mixed ANOVA with the standardized individual estimates of the coefficients as a dependent variable. We found a significant effect by class interaction ($F_{(1,176)}$=295.968, p<.001, η_g^2=.627, *Figure S9*), showing that the SR class had a larger standardized coefficient of the intercept than the condition effect (emm=-2.24, p_{bonf}<.001), whereas the opposite was true for the GD class (emm=1.06, p_{bonf}<.001; *Figure S7*). Following the reasoning that the intercept is a trace of SR processes and that the condition effect is a trace of GD processes, we could conclude that avoidance was mostly driven by SR processes in the SR class and GD processes in the GD class. Interestingly, we further found that the condition effect in the SR class was not significantly different from the intercept estimate in the GD class (emm=.155, p_{bonf}=1, $p_{uncorrected}$=.253), suggesting that avoidance in the GD class was purely GD, and that avoidance in the SR class was purely SR. Finally, the result that the estimate of the intercept in the SR class was significantly larger than the estimate of the condition effect in the GD class (emm=-1.018, p_{bonf}<.001) could explain the higher avoidance rate in the SR class (i.e., SR processes are more active here than GD processes in the GD class). This confirms the results of the odds ratios of the two effects presented in the main text, which reveal an intercept non-credibly different from 0 and a condition effect credibly above 0 in the GD class, and the opposite pattern in the SR class.

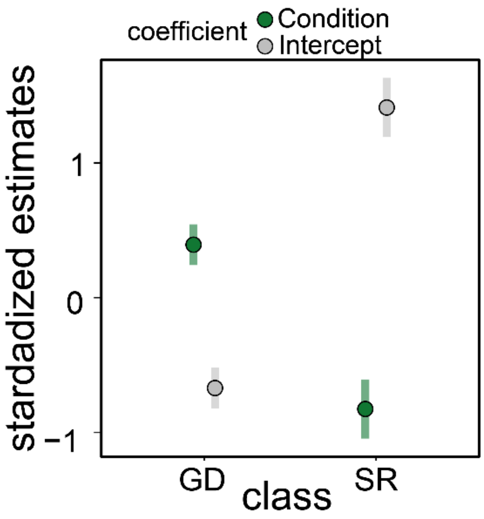

Figure S9: Group-level estimates of standardized coefficients estimates*. Point: Median posterior estimate. Vertical lines: 95%CrI. Goal-Directed (GD) class: n=69, Stimulus-Response (SR) class: n=31.*

Supplementary Note 7: Metanalytic analyses

To test the reliability of DDM parameters of interest across the three experiments, we conducted a Bayesian random effects meta-analysis on our results using the R-package brms[5]. We opted for a Bayesian approach for meta-analysis as it has been shown to outperform frequentist ones when a limited number of studies is available[12]. Bayesian models have been fitted through 4 chains of 30,000 iterations each (15,000 used as warm up period) and a thinning factor of 10. We used a Normal(0,1) prior for the mu parameter and a HalfCauchy(0, 0.5) prior for the standard deviation parameter. The analyses have been run on parameters' estimates from the three experiments and the relative measurement error computed as (upper limit - lower limit of parameters' estimates 95%CrI) / 3.92. To be able to compare the three experiments, for Experiment 2 we used parameters from a DDM fitting the Condition effect on drift rate, boundary separation and non-decision time, even though this was not the best fitting model in this experiment.

When testing the drift rates, we found credible meta-analytic effects both for the intercept (i.e., drift rate in the Unpredictable condition: estimate=.408; 95%CrI=[.013, .738]; $p_{<0}$= .023; R-hat=1.001; ESS=5055) and for the effect of Predictability (estimate=.275; 95%CrI=[.009, .545]; $p_{<0}$= .024; R-hat=1.001; ESS=5053; *Figure S10*). Conversely, we did not find credible metanalytic effects for the effect of Condition neither on the boundary separation (estimate=.027; 95%CrI=[-.095, .136]; $p_{<0}$=.177; R-hat=1; ESS=4656) nor on the non-decision time (estimate=.001; 95%CrI=[-.069, .076]; $p_{<0}$= .485; R-hat=1; ESS=3757).

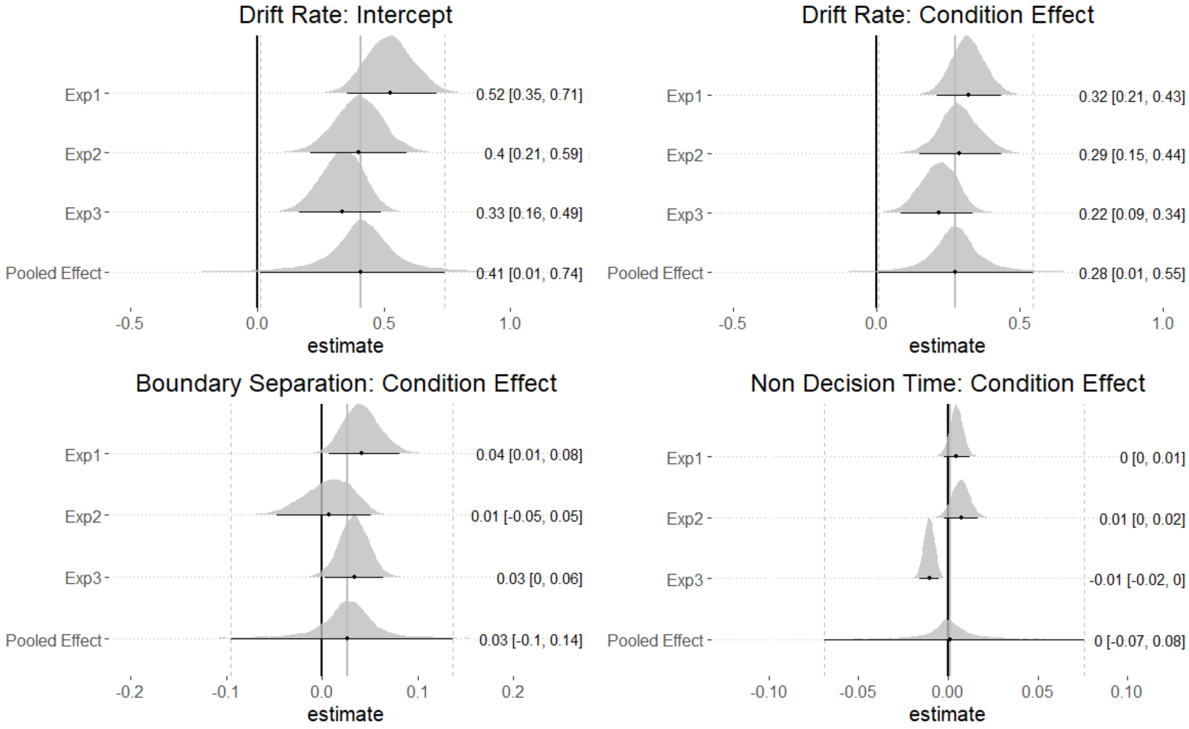

***Figure S10**: Forest plots for each meta-analytic effect. Density distributions: posterior densities for parameter estimates across the three experiments and pooled effect. Vertical thin lines: median (solid line) and 95%CrI (dashed lines) of pooled effect distribution. Numbers on the right: posterior median and 95%CrI (in brackets) estimate for each parameter. Note that the parameters reported in the figure for the three experiments are the shrinked parameters after hierarchical meta-analytical estimation. Exp: Experiment.*

Supplementary Note 8: Head-Tracking

The Unity-UXF interface[11] provides a continuous measure of head rotation for each trial on the x, y and z axis (i.e., pitch, roll and yaw rotation values) sampled at 60 frames per second. The rotation measurement thus extracted represents participants' fixation and is centered at the center of the visual scene (i.e., values of x and z are zero if participants orient their head in front of them with a 90° angle from their torso) regardless of the position in the virtual environment. In order to explore participants' visual exploration in relation to avatars' faces during the trial, we computed a continuous measure of rotation toward or away the face of the avatar in the chosen elevator in a given trial.

Data preprocessing and analysis

Initially, we define a 'fixation point' within the 3D environment by the x and z coordinates of the camera (i.e., the moving participants' position over time) and the y coordinate of the avatar's face in the chosen elevator. The 'distance of fixation' was then defined as the horizontal distance between the camera and this fixation point. Subsequently, we employed the tangent function to calculate the frame-by-frame displacement of the fixation point from its original position depending on participants' head orientation on the horizontal and vertical axes. This computation allowed for a continuous update of the fixation point. Next, we computed three sides of a two-dimensional triangle within a three-dimensional space. These sides included the Euclidean distances between the avatar's face in the chosen elevator, the fixation point, and the camera. With these distances, we could then calculate frame by frame the angle between the participants' fixation point and the avatar's face using Carnot's theorem. Finally, since head rotation from avatars varied also depending on the camera's automatic movement in space (i.e., when entering the elevator), we adjusted these frame-by-frame measures by subtracting the hypothetical angle that participants would have presented assuming no movement. This adjustment yielded a measure of head movement in degrees, indicating whether participants moved their head away (positive values) or toward (negative values) the face in the chosen elevator.

The resulting head rotation time series were analyzed using the one-dimensional statistical parametric mapping approach used for physiological data in Experiment 3 and described in the main text of the manuscript (*Experiment 3 - Material and Method - Data Analysis -*

Physiological data). Before fitting the GLM, data were z-scored within participants using mean and standard deviation of the concatenated trial data.

Results

In all experiment we found a significant negative cluster for the intercept at the end of the trial during the outcome phase (Intercept - Experiment 1: t_{mass}=-3131.9, p_{cor}= 0; Experiment 2: t_{mass}=-1427.3; p_{cor}=.032; Experiment 3: t_{mass}=-2134.2; p_{corr}=.001; *Figure S11*). A negative cluster indicates that participants reduced the angle of head orientation from the face in the chosen elevator, viz they looked at the avatar. This result shows that regardless of the outcome of the choice, the condition and the RT, participants oriented their head (and likely their attention) toward the face of the avatar, thus receiving the intended feedback (i.e. the emotional expression randomly changing in the Unpredictable condition).

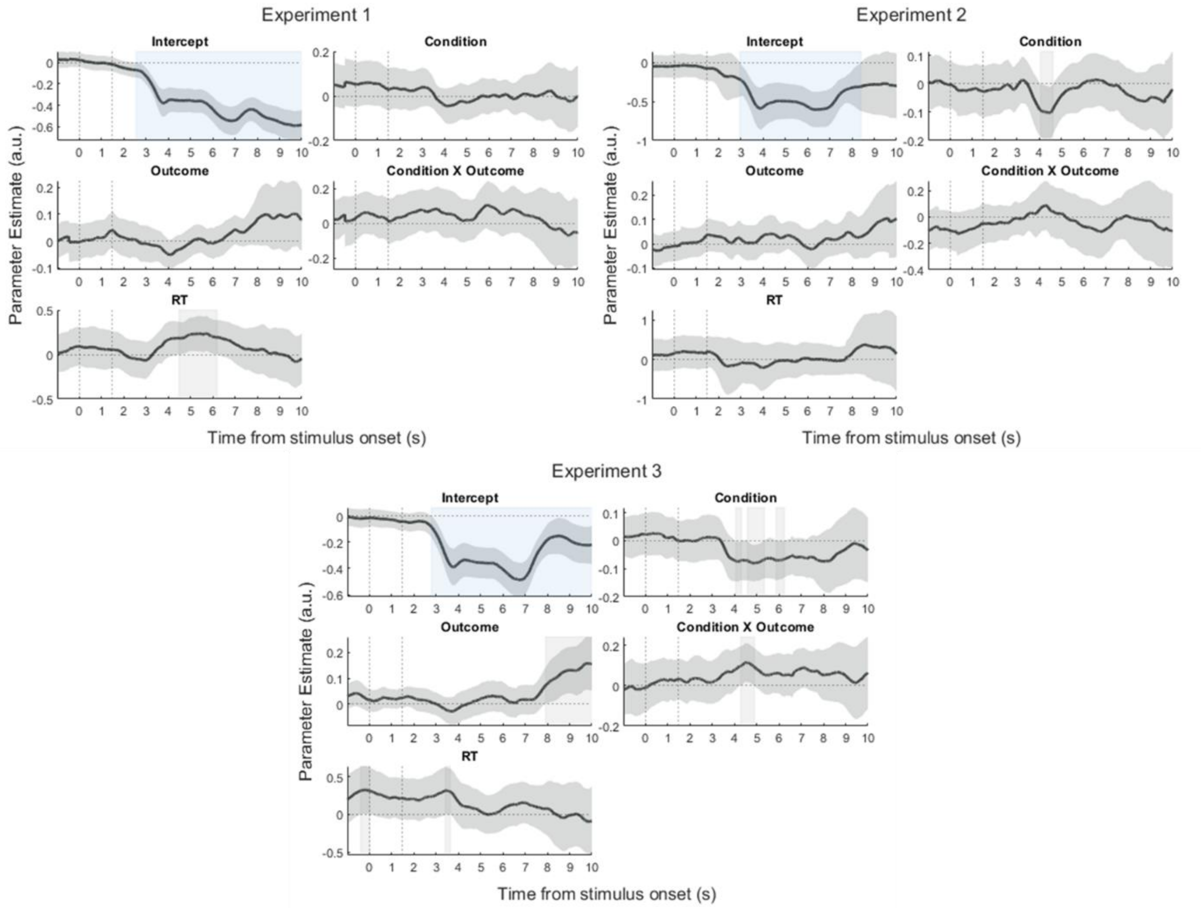

Figure S11: GLM results on head rotation from the chosen face for each experiment. *Positive values: looking away from the face of the avatar in the chosen elevator. Negative values: looking toward the face of the avatar in the chosen elevator. Vertical dashed lines: doors' opening and end of the choice time. Shaded areas: 95% Confidence Interval. Blue and grey rectangles areas: significant and non-significant clusters. Experiment 1: n=60,*

Experiment 2: n=30, Experiment 3: n=60. a.u.: Arbitrary Unit. RT: Response Time.

Supplementary Discussion

Effect of Response Times on avoidance rate

Across the three experiments, the probability of avoidance in both conditions depended on response times, with faster RTs associated with avoidance at or near chance. The fact that faster RTs were associated with chance level avoidance rate in this task may reflect random responses, as participants were instructed to choose as quickly as possible, while maintaining fixation on the central arrow. In other words, the random avoidance rate at this short latency seems to be due to a speed-value trade-off[14].

Interestingly, whereas in Experiment 1, the difference between conditions was independent of RTs, in Experiment 2 and 3, we found that this difference was more evident with slower RTs, as reflected by a condition by RTs interaction. Note that in Experiment 1, participants only had to accumulate evidence for the location of the threatening/neutral expressions, as they already had the time to process the cue indicating the predictable/unpredictable condition before the stimulus onset. Consequently, even when responding rapidly they may have had time to combine the information about outcome (un)predictability with threat location. Under these circumstances, even the faster RTs on the distribution would be slow enough to allow for the condition effect to manifest itself (see *Figure 1D* in the main manuscript), making the condition by RTs interaction less likely to emerge.

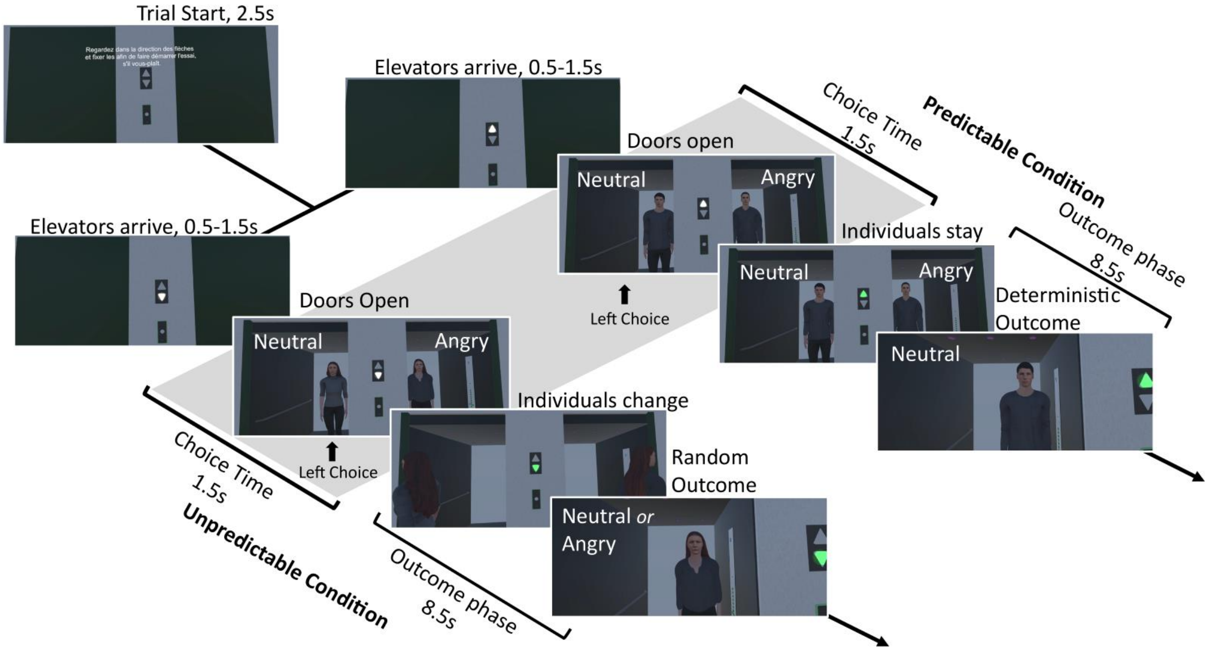

Figure S12. Task design in Experiment 1. *In Experiment 1, a trial of the Elevator Task began with a screen that required fixation on the central arrows (2.5s). Then one of the two arrows lit up, indicating whether the elevators were going up or down (anticipating avatars' movements after the choice). After a jittered interval, the two doors opened simultaneously, and participants had 1.5s to freely choose which elevator to enter (by pressing the left or the right button). After the choice, in the Predictable condition (up arrow lit), participants were led next to the avatar in the chosen elevator during a period lasting 8.5s (4s moving inside the elevator, 4.5s standing next to the avatar). In the Unpredictable condition (down arrow lit), after participants made their choice, the avatars in the elevators walked outside the environment, while two new different avatars (not previously visible) walked inside the elevators. Thus, participants were led next to a new avatar (50% probability of being angry or neutral). At the end of the trial, participants were "teleported" back to the starting position. The scene reset between two trials in 0.5 seconds. Note that the "Angry" and "Neutral" texts are for illustrative purposes only and were not present in the virtual environment.* The picture was created as an illustrative example of the task: avatars' faces were modeled from the Radboud face database[1], and the 3D body models were purchased from the RenderPeople website (https://renderpeople.com). Both sources permit the use of stimuli as examples.

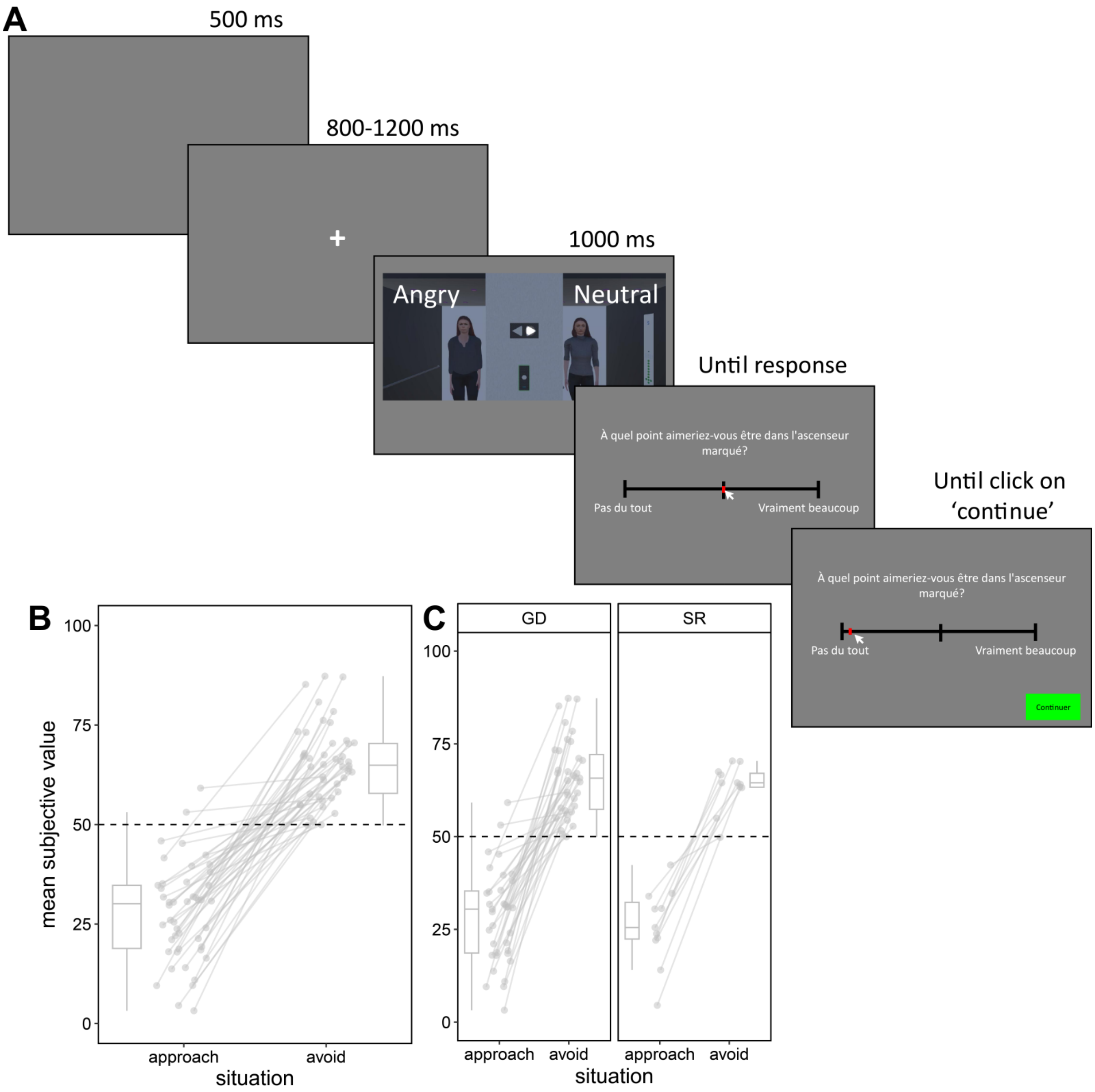

Figure S13: Subjective evaluation task. *(A) In the subjective evaluation task that followed the VR task, participants had to subjectively evaluate each possible choice scenario seen during the main task (the example represents an avoidance scenario). Note that the "Angry" and "Neutral" texts are for representational purposes only and were not present in the task.* The picture was created as an illustrative example of the task: avatars' faces were modeled from the Radboud face database[1], and the 3D body models were purchased from the RenderPeople website (https://renderpeople.com). Both sources permit the use of stimuli as examples. *(B) Participants consistently preferred to avoid the angry avatar compared to approach it, with no difference between the two latent classes (n=46) (C). Single points represent individual participants. Boxplots represent first, second (median) and third quartile. Whiskers are drawn within the 1.5 interquartile range.* GD: Goal-Directed class. SR: Stimulus-Response class.

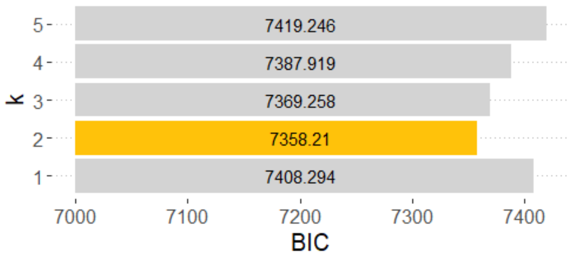

Figure S14: Finite Mixture Model comparison. *Model comparison results for finite mixture modeling. K: number of components. BIC: Bayesian Information Criterion. Yellow: best fitting number of components (i.e., lowest BIC). N=90.*

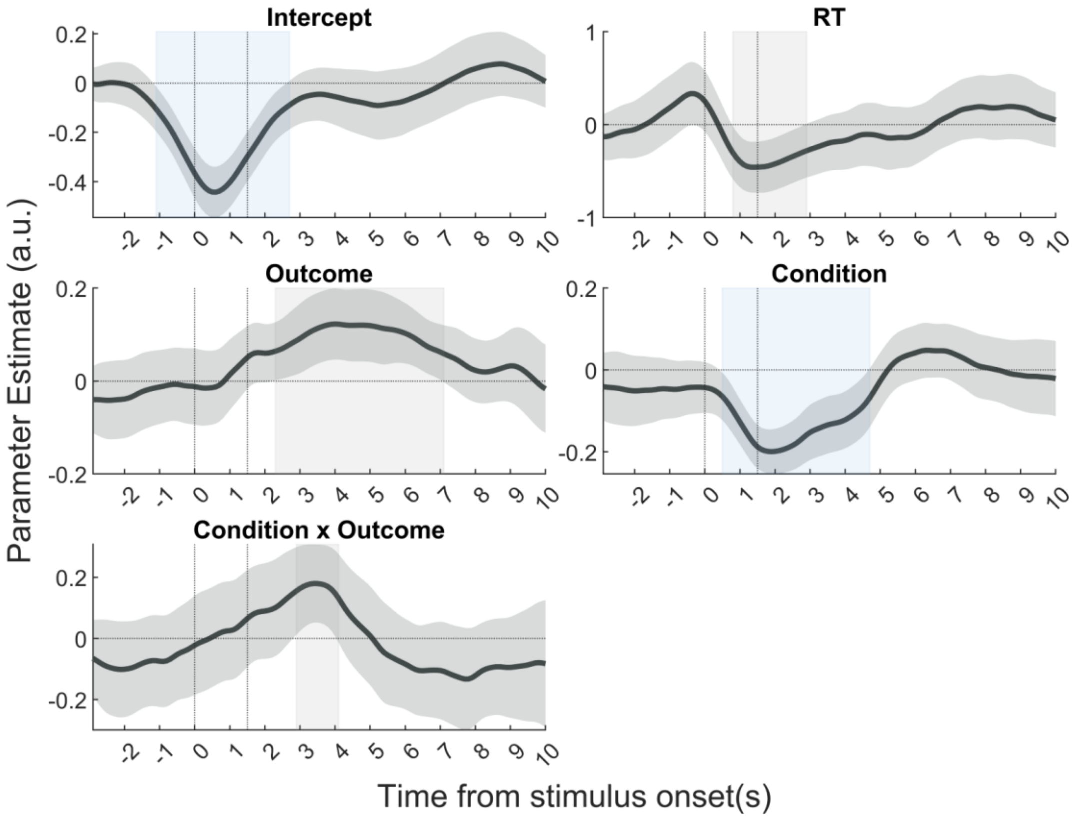

Figure S15: GLM results on iHR in Experiment 3. *Blue shaded rectangles are significant clusters, Grey rectangles are non-significant clusters. Thick lines: parameter estimate. Shaded areas: 95% confidence interval. Dotted vertical lines: Stimulus onset (i.e. doors' opening) and end of the response period. N=55.* a.u.: Arbitrary Unit. RT: Response Time.

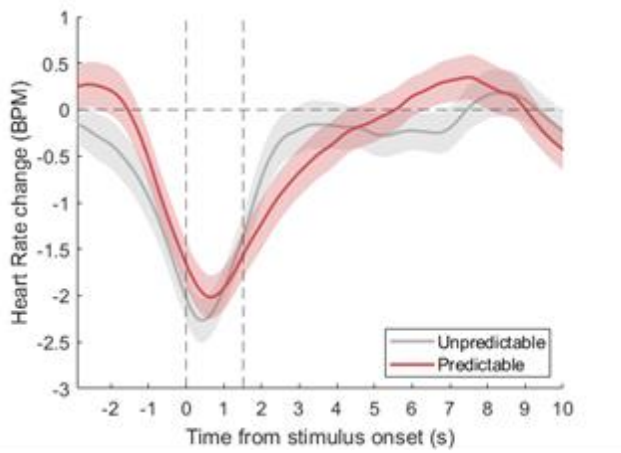

Figure S16: ECG activity in neutral trials. *Grand Average iHR series (change from baseline in BPM, Beats Per Minute) by Condition in neutral trials of Experiment 3. Dashed vertical Lines: Stimulus onset (i.e. doors' opening) and end of response period. Shaded areas: 95% within subject confidence intervals. N=55.*

Table S47: *Sample description and Self-reported measures across experiments.*

	**Experiment 1 (n=60)**		**Experiment 2 (n=30)**		**Experiment 3 (n=60)**	
	Stat	Cronbach α	Stat	Cronbach α	Stat	Cronbach α
Age	23 [18, 35]		22 [18, 33]		22 [18, 35]	
Sex	30F/30M		15F/15M		32F/28M	
Handedness	6L/53R/1A		3L/27R		4L/55R/1A	
Education	6A/11B/43C		4A/4B/22C		14A/5B/41C	
LSAS	37.0 [6, 91]	.91	32.5 [0, 63]	.90	44.5 [2, 85]	.94
STAI-S	28.0 [20, 50]	.89	32.0 [20, 51]	.89	29.0 [20, 47]	.81
STAI-T	42.0 [24, 73]	.90	41.5 [28, 64]	.87	43.0 [27, 64]	.84
BIS	21.0 [12, 26]	.68	20.5 [12, 28]	.80	20.0 [14, 26]	.60
BAS-DR	9.0 [5, 13]	.59	9.0 [6, 14]	.60	9.0 [5, 14]	.56
BAS-FS	12.0 [7, 16]	.55	13.0 [8, 16]	68	12.0 [7, 16]	.64
BAS-RR	17.0 [11, 19]	.55	17.0 [12, 19]	.51	17.0 [11, 20]	.52
PHQ-8	4.0 [0, 15]	.69	5.5 [0, 12]	.70	6.0 [0, 19]	.75
PANAS-P	33.0 [18, 44]	.88	33.5 [25, 42]	.70	33.0 [14, 43]	.77
PANAS-N	16.0 [10, 40]	.81	17.0 [10, 33]	.84	17.5 [10, 35]	.84
Cybersickness	7.0 [0, 26]	.81	6.0 [0, 30]	.90	8.5 [0, 25]	.76

Note: Stat: descriptive statistic, median [min, max] for continuous variables, count for categorical variables. Handedness: L: left handed, R: right handed, A: Ambidextrous. Education: A: high-school level education, B: two years of education after high-school, C: more than two years of education after high-school. LSAS: Liebowitz Social Anxiety Scale. STAI-S and -T: State and Trait subscales of the State-Trait Anxiety Inventory. BIS: Behavioral Inhibition Scale. BAS-DR, -FS and -RR: Behavioral Approach Scale, Drive, Fun Seeking and Reward Responsiveness subscales. PHQ-8: Patient Health Questionnaire 8. PANAS-P and -N: Positive and Negative Affective Scale, Positive and Negative subscales. Cybersickness: simulator sickness questionnaire.

Table S48: *Proportion of avoidance responses and Response Times for all experiments.*

		**P(Avoid angry)**		**Approach RT (ms)**		**Avoid RT (ms)**	
	condition	Prop	SD	median	MAD	median	MAD
**Exp1 (n=60)**	Unpred.	.647	.478	557.133	330.738	752.044	309.882
	Pred.	.726	.446	529.169	310.258	772.930	278.480
**Exp2 (n=30)**	Unpred.	.599	.490	543.341	371.164	682.385	392.671
	Pred.	.659	.474	515.276	330.376	766.066	350.532
**Exp3 (n=60)**	Unpred.	.574	.495	473.414	268.142	598.837	371.420
	Pred.	.613	.487	445.221	227.364	668.103	351.250

Note: RT: Response Time. Unpred. and Pred.: Unpredictable and Predictable condition. Prop: Proportion of avoidance choices. SD: Standard Deviation. MAD: Median Absolute Dispersion.

Supplementary References

1. Langner, O. *et al.* Presentation and validation of the Radboud Faces Database. *Cogn. Emot.* 24, 1377–1388 (2010).
2. Feng, Y., Feng, H., Black, M. J. & Bolkart, T. Learning an Animatable Detailed 3D Face Model from In-The-Wild Images. Preprint at http://arxiv.org/abs/2012.04012 (2021).
3. Cignoni, P., Corsini, M. & Ranzuglia, G. MeshLab: an Open-Source 3D Mesh Processing System.
4. Treal, T., Jackson, P. L., Jeuvrey, J., Vignais, N. & Meugnot, A. Natural human postural oscillations enhance the empathic response to a facial pain expression in a virtual character. *Sci. Rep.* 11, 12493 (2021).
5. Bürkner, P.-C. brms : An R Package for Bayesian Multilevel Models Using Stan. *J. Stat. Softw.* 80, (2017).
6. McElreath, R. *Statistical Rethinking: A Bayesian Course with Examples in R and Stan*. (Chapman and Hall/CRC, 2018).
7. Mennella, R., Vilarem, E. & Grèzes, J. Rapid approach-avoidance responses to emotional displays reflect value-based decisions: Neural evidence from an EEG study. *NeuroImage* 222, 117253 (2020).
8. Grèzes, J. *et al.* Impact of total sleep deprivation and related mood changes on approach-avoidance decisions to threat-related facial displays. *Sleep* 44, zsab186 (2021).
9. Wiecki, T., Sofer, I. & Frank, M. HDDM: Hierarchical Bayesian estimation of the Drift-Diffusion Model in Python. *Front. Neuroinformatics* 7, (2013).
10. Matzke, D. & Wagenmakers, E.-J. Psychological interpretation of the ex-Gaussian and shifted Wald parameters: A diffusion model analysis. *Psychon. Bull. Rev.* 16, 798–817 (2009).
11. Brookes, J., Warburton, M., Alghadier, M., Mon-Williams, M. & Mushtaq, F. Studying human behavior with virtual reality: The Unity Experiment Framework. *Behav. Res. Methods* 52, 455–463 (2020).
12. Reis, D. J. *et al.* A Practical Guide to Random-Effects Bayesian Meta-Analyses With Application to the Psychological Trauma and Suicide Literature. *Psychol. Trauma Theory Res. Pract. Policy* 15, 121–130 (2023).
13. Lenth, R. V. emmeans: Estimated Marginal Means, aka Least-Squares Means. 1.10.4 https://doi.org/10.32614/CRAN.package.emmeans (2017).
14. Pirrone, A., Stafford, T. & Marshall, J. A. R. When natural selection should optimize speed-accuracy trade-offs. *Front. Neurosci.* 8, (2014).
