# Supplementary material for: Social threat avoidance depends on action-outcome predictability
Source: Commun Psychol. 2024 Oct 26;2:100. doi: 10.1038/s44271-024-00152-y (PMC11512816; doi:10.1038/s44271-024-00152-y)
Supplement: Supplementary file 3 — Reporting Summary [file 44271_2024_152_MOESM3_ESM.pdf]

nature portfolio

Corresponding author(s): Matteo Sequestro, Julie Grèzes

Last updated by author(s): Oct 9, 2024

Reporting Summary

Nature Portfolio wishes to improve the reproducibility of the work that we publish. This form provides structure for consistency and transparency in reporting. For further information on Nature Portfolio policies, see our Editorial Policies and the Editorial Policy Checklist.

Statistics

For all statistical analyses, confirm that the following items are present in the figure legend, table legend, main text, or Methods section.

n/a	Confirmed	
☐	☒	The exact sample size (*n*) for each experimental group/condition, given as a discrete number and unit of measurement
☐	☒	A statement on whether measurements were taken from distinct samples or whether the same sample was measured repeatedly
☐	☒	The statistical test(s) used AND whether they are one- or two-sided<br>*Only common tests should be described solely by name; describe more complex techniques in the Methods section.*
☐	☒	A description of all covariates tested
☐	☒	A description of any assumptions or corrections, such as tests of normality and adjustment for multiple comparisons
☐	☒	A full description of the statistical parameters including central tendency (e.g. means) or other basic estimates (e.g. regression coefficient) AND variation (e.g. standard deviation) or associated estimates of uncertainty (e.g. confidence intervals)
☐	☒	For null hypothesis testing, the test statistic (e.g. *F*, *t*, *r*) with confidence intervals, effect sizes, degrees of freedom and *P* value noted<br>*Give P values as exact values whenever suitable.*
☐	☒	For Bayesian analysis, information on the choice of priors and Markov chain Monte Carlo settings
☐	☒	For hierarchical and complex designs, identification of the appropriate level for tests and full reporting of outcomes
☐	☒	Estimates of effect sizes (e.g. Cohen's *d*, Pearson's *r*), indicating how they were calculated

Our web collection on statistics for biologists contains articles on many of the points above.

Software and code

Policy information about availability of computer code

Data collection	We collected data by a Virtual Environment developed using the Unity game engine (version 2020.3.19f1, Unity Technologies) and the UXF package (Unity Experiment Framework; Brookes et al., 2020).
Data analysis	We used the HDDM package (v0.9.7) in Python (3.7.13) to fit Drift Diffusion Model. We used custom code in MATLAB (R2022b) to process and analyze physiological data. We used R (v4.1.1) and RStudio (v2023.6.2.561) to perform statistical analyses. In particular, we used the R package brms (v2.16.1) to fit hierarchical bayesian regression models.

For manuscripts utilizing custom algorithms or software that are central to the research but not yet described in published literature, software must be made available to editors and reviewers. We strongly encourage code deposition in a community repository (e.g. GitHub). See the Nature Portfolio guidelines for submitting code & software for further information.

Data

Policy information about availability of data

All manuscripts must include a data availability statement. This statement should provide the following information, where applicable:

- Accession codes, unique identifiers, or web links for publicly available datasets
- A description of any restrictions on data availability
- For clinical datasets or third party data, please ensure that the statement adheres to our policy

All data are freely available at the following OSF repository: https://osf.io/rucz9/

Human research participants

Policy information about [studies involving human research participants and Sex and Gender in Research.]

Reporting on sex and gender	Participants self-reported information about sex (biological attribute) and provided consent for sharing this info. While sex was not considered in our study design, we ran control analyses to explore the effect of participants' sex on our result and found no difference between female and male participants (Supplementary Material L), thus showing generalizability.
Population characteristics	The study included three experiments with independent samples [Experiment 1: n=60, 30 females, mean age (sd)=23.51 (3.83); Eperiment 2: n=30, 15 females, age=22.60 (3.29); Experiment 3: n=60, 32 females, age=23.42 (4.59)]. See Supplementary Table G2 for a detailed description of the samples.
Recruitment	Participants were recruited by convenience sampling through flyers on the university campus and online advertisement. Even though our three samples were perfectly balanced in terms of participants' sex, still participants were sampled from a "WEIRD" population, and this study should be replicated on a broader population.
Ethics oversight	The experimental protocol for the experiments was approved by INSERM, licensed by the local research ethics committee (IRB00003888 – Avis 18-544-ter - 25.10.2021).

Note that full information on the approval of the study protocol must also be provided in the manuscript.

Field-specific reporting

Please select the one below that is the best fit for your research. If you are not sure, read the appropriate sections before making your selection.

☐ Life sciences ☒ Behavioural & social sciences ☐ Ecological, evolutionary & environmental sciences

For a reference copy of the document with all sections, see nature.com/documents/nr-reporting-summary-flat.pdf

Behavioural & social sciences study design

All studies must disclose on these points even when the disclosure is negative.

Study description	The study included quantitative behavioral data collected in a Virtual Reality task studying approach-avoidance decision making in a socioemotional context. The study also involved quantitative self-report measures (depression, axiety, appetitive and aversive motivation, cybersickness symptoms, positive and negative affect) and physiological (ECG, EMG) recordings.
Research sample	Participants were aged between 18 and 35 years old and had no history of neurological or psychiatric disorders. A full list of inclusion criteria is provided in Supplementary Material B. Additional details regarding sample size, sex, age and recruitment methods are given above (see Human Research Participants).
Sampling strategy	The sample was recruited using convenience sampling through flyers on the university campus and online advertisements. The sample size was chosen by a power simulation approach (Kumle et al., 2021) on a pilot experiment for Experiment 1, and on the previous experiment for Experiments 2 and 3.
Data collection	Behavioral data was collected through a Virtual Environment developed using the Unity game engine (version 2020.3.19f1, Unity Technologies) and the UXF package (Unity Experiment Framework; Brookes et al., 2020). The Virtual Environment was presented through the Virtual Reality Head Mounted Display Oculus Quest 2 (Meta Quest). Two Oculus Touch Controllers (one per hand) were used to collect participant responses. The software was run on an Alienware Aurora Ryzen Edition PC with a 3.00GHz 12-core AMD Ryzen 9 5900 processor, a 32GB DDR4 3400 MHz RAM, and NVIDA GeForce RTX 3080 10GB GDDR6X graphic card. Self-report measure were administered either online through Qualtrics (Qualtrics, Provo, UT) or on site through a laptop. Physiological data were collected using ADInstruments hardware (ADInstrument, Amsterdam, NL) and amplified prior to digitalization (Dual BioAmp, ADInstrument). The Subjective Evaluation Task was implemented in MATLAB (R2022b) using Psychtoolbox (v3.0.18).
Timing	Data for Experiment 1 was collected between July and October 2022. Data for Experiment 2 was collected between December 2022 and January 2023. Data for Experiment 3 was collected between April and June 2023.
Data exclusions	In Experiment 1 we excluded data from one participant due to cybersickness symptoms during the task and for one participant who reported to be already familiary with the task. In Experiment 2 we excluded data from one participant due to cybersickness during the task symptoms and for one participant who showed a lack of understanding of the task's instructions. In Experiment 3 we excluded data from 3 participants due to cybersickness symptoms during the task.
Non-participation	No participants dropped out/declined participation.
Randomization	Participants were not allocated into experimental groups.

Reporting for specific materials, systems and methods

We require information from authors about some types of materials, experimental systems and methods used in many studies. Here, indicate whether each material, system or method listed is relevant to your study. If you are not sure if a list item applies to your research, read the appropriate section before selecting a response.

Materials & experimental systems

n/a	Involved in the study
☒	☐ Antibodies
☒	☐ Eukaryotic cell lines
☒	☐ Palaeontology and archaeology
☒	☐ Animals and other organisms
☒	☐ Clinical data
☒	☐ Dual use research of concern

Methods

n/a	Involved in the study
☒	☐ ChIP-seq
☒	☐ Flow cytometry
☒	☐ MRI-based neuroimaging
